# Fatty acid metabolism: opportunities and challenges of traditional Chinese medicine in the treatment of renal fibrosis

**DOI:** 10.1186/s13020-026-01328-w

**Published:** 2026-01-21

**Authors:** Ran-ran Gao, Cong Han, Wei Li

**Affiliations:** 1 The First Clinical College, Shandong University of Traditional Chinese Medicine, Jinan, China; 2 Nephropathy Department, Affiliated Hospital of Shandong University of Traditional Chinese Medicine, Jinan, China

**Keywords:** Renal fibrosis, Fatty acid metabolism, Traditional Chinese medicine, Multiple targets, Mitochondrial dysfunction, Lipotoxicity

## Abstract

Renal fibrosis (RF), as a critical pathological process in the progression of chronic kidney disease (CKD) to end-stage renal disease, currently lacks clear and effective reversal measures. Disordered fatty acid metabolism is highly involved in this process. Studies have revealed that molecular crosstalk under fatty acid metabolism dysregulation—such as lipotoxicity, mitochondrial damage, immune inflammation, epigenetics, metabolic reprogramming, and the gut-kidney axis—drives damage to renal tubules, glomeruli, and the interstitium. Therapeutic strategies targeting fatty acid metabolism to ameliorate RF have gradually become a research hotspot, though most current drugs are still in the clinical exploration stage, and the predictive and therapeutic value of many biomarkers remains to be validated. Traditional Chinese Medicine (TCM), with its personalized and modernized approach combining oral administration and external treatments such as acupuncture, enema, and nanodelivery, is increasingly demonstrating advantages in targeting fatty acid metabolism to improve RF. This review outlines the mechanisms and core targets of TCM in regulating fatty acid metabolism to ameliorate RF. The multi-target advantage based on complex components presents an opportunity for TCM to improve RF, though current research still faces challenges such as mechanistic complexity, standardization of TCM, and clinical translation. Future efforts should integrate multi-omics technologies, novel drug delivery systems, and disease-syndrome combination models to advance the development of new integrated Chinese and Western medicine drugs for RF.

**Graphical abstract:**

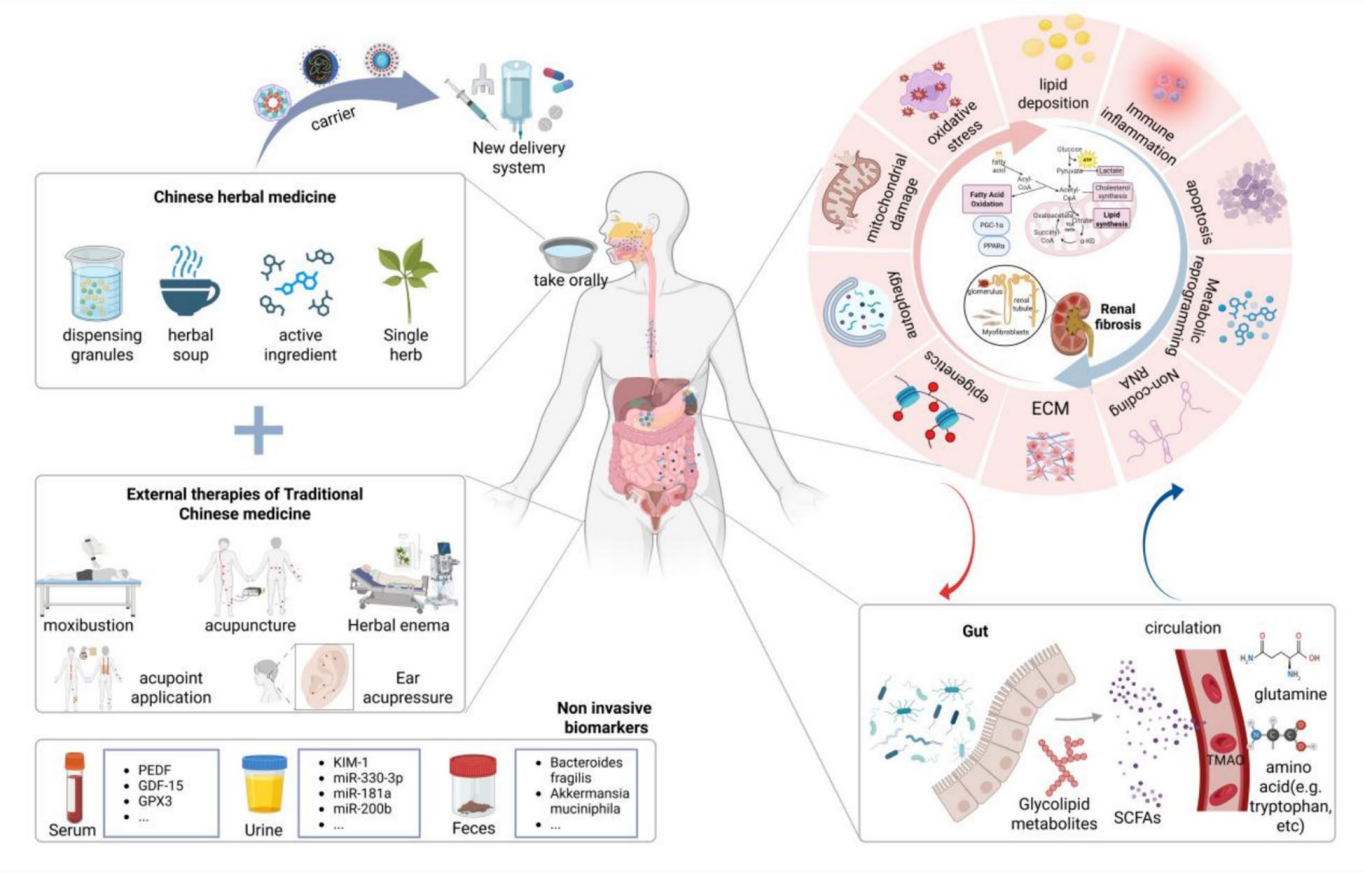

## Key points

1. Disordered fatty acid metabolism plays a significant role in the process of renal fibrosis and is a key mechanism in the development and progression of RF.
2. Molecular crosstalk under fatty acid metabolism dysregulation—such as lipotoxicity, mitochondrial damage, immune inflammation, epigenetics, metabolic reprogramming, and the gut-kidney axis—drives damage to renal tubules, glomeruli, and the interstitium.
3. Traditional Chinese Medicine (TCM), with its personalized and modernized approach combining oral administration and external treatments like acupuncture, enema, and nanodelivery, targets fatty acid metabolism to improve RF, offering unique advantages such as a multi-targeted approach, reduced toxicity, enhanced efficacy, and minimal side effects.
4. The multi-target advantage based on complex components presents an opportunity for TCM to ameliorate RF, though current research still faces challenges such as mechanistic complexity, standardization of TCM formulations, and clinical translation.
5. The integration of traditional Chinese medicine with modern medical technology can break through developmental limitations and play a crucial role in the treatment of RF in the future.

## Introduction

Renal fibrosis (RF) is the pathological basis for the progression of chronic kidney disease (CKD) to end-stage renal disease (ESRD), and it often occurs alongside the progression of CKD. Globally, more than 10% of the population suffers from CKD, which has now become a significant public health concern [1]. Once patients enter the ESRD phase, they often require renal replacement therapy, which imposes a substantial economic and social burden. Therefore, understanding the mechanisms underlying the occurrence and development of RF and delaying its progression are highly important.

The pathological features of RF are characterized mainly by glomerulosclerosis, renal tubular atrophy, chronic interstitial inflammation, fibrosis, and vascular rarefaction [2]. Its pathogenesis has not been fully elucidated. Relevant studies have shown that it is associated mainly with conditions such as inflammatory activation and immune cell infiltration, the release of profibrotic mediators, excessive deposition of the extracellular matrix (ECM), damage to intrinsic renal cells, and a reduction in the renal microvascular system [3]. In recent years, it has been increasingly recognized that lipid metabolism disorders can cause mitochondrial and renal cell damage through activation of the innate immune system and inflammation, especially imbalances in mitochondrial energy metabolism, thereby promoting RF, which is a major cause of the progression of CKD [4–7]. Currently, there are no effective drugs that specifically target RF. Renin-angiotensin system (RAS) blockers [8], sodium‒glucose cotransporter 2 (SGLT2) inhibitors [9], glucagon-like peptide-1 (GLP-1) receptor agonists [10], nonsteroidal mineralocorticoid receptor antagonists (nsMRAs) [11] and nonsteroidal antiminocorticoids [12] can delay the progression of CKD and improve RF to varying degrees [13, 14]. However, their efficacy is limited, and there are risks such as elevated creatinine levels. Despite continuous focused research on the mechanisms and therapeutic targets of RF, no new safe and effective targeted drugs have been applied in clinical practice to date [15, 16]. Therefore, it is particularly important to explore safe and effective methods for the prevention and treatment of RF.

In traditional Chinese medicine (TCM), RF can be categorized into categories such as "edema" and "turbid urine". Its fundamental pathogenesis can be summarized as deficiency in origin and excess in superficiality: deficiency in origin mainly lies in spleen-kidney qi deficiency, leading to dysfunction of transportation, transformation, and qi transformation; excess in superficiality results from the accumulation of pathological products including dampness, turbidity, blood stasis, and toxin in the renal collaterals [17]. Chinese herbal medicines have gradually shown certain advantages in improving RF. Numerous clinical and basic experimental studies have demonstrated that Chinese herbal medicines and their active ingredients can ameliorate RF and delay the progression of CKD [18–22]. Modern studies have indicated that qi deficiency and blood stasis are closely related to mitochondrial energy metabolism [23]. Dampness-turbidity is highly associated with lipid metabolism disorders and accumulation of inflammatory mediators [24]. While blood stasis corresponds to microcirculation dysfunction and abnormal deposition of ECM [25, 26]. Notably, in TCM theory, the normal operation of qi transformation depends on material basis, and modern medical research has confirmed that the kidney is a high-energy-consuming organ whose main energy source is precisely mitochondrial fatty acid oxidation. Therefore, the homeostasis of renal energy metabolism, especially fatty acid metabolism, serves as an essential material basis for maintaining normal qi transformation and preventing internal generation of dampness-turbidity. Regulating lipid production, transportation and decomposition functions with drugs to improve lipid metabolism disorders, especially fatty acid metabolism disorders, and regulating renal energy metabolism can be used as effective methods to prevent and treat RF and delay the progression of CKD [27], which has attracted extensive attention in recent years. At present, various Chinese herbal medicines and their active ingredients have been found to affect lipid metabolism and ameliorate fatty acid metabolism disorders.

Therefore, in this article, we review the relationships between fatty acid metabolism and RF, as well as Chinese medicinal herbs and their monomers targeting fatty acid metabolism for the treatment of RF, and integrates TCM pathogenesis and therapeutic principles with Western targets and pathways to clarify their scientific connotation. Specifically, we summarize the different mechanisms by which fatty acid metabolism participates in RF in different renal structures, and how Chinese medicine exerts its effects around these targets under pathological conditions. We also organically integrate traditional Chinese and Western medicine, and interpret TCM macroscopic pathogenesis and therapeutic principles using modern microscopic theories. This study is expected to provide new ideas for the mechanistic research and clinical intervention of RF, delay the progression of RF, and promote the development of TCM.

## Methods

In this review, we conducted a systematic and comprehensive retrieval of relevant literature published in authoritative databases such as PubMed (https://pubmed.ncbi.nlm.nih.gov), Web of Science (http://apps.webofknowledge.com/), and CNKI (http://www.cnki.net) up to December 2025. The keywords included "renal fibrosis", "chronic kidney disease", "fatty acid metabolism", "lipid metabolism", "Chinese herbal medicine", "natural products", "active compounds", "Chinese medicinal preparations", "traditional Chinese medicine", "acupuncture", "Chinese medicine enema", and "nanoparticles", among others. The inclusion criteria focused on therapies targeting fatty acid metabolism for renal fibrosis, especially the latest findings on traditional Chinese medicine targeting fatty acid metabolism in the treatment of renal fibrosis. This review has no language restrictions, comprehensively includes all published studies in Chinese and English, and fully considers in vitro, in vivo, and clinical trials. During the process of literature screening, we followed the principles of rigor and order.

## Fatty acid metabolism and renal fibrosis

**Fig. 1:**
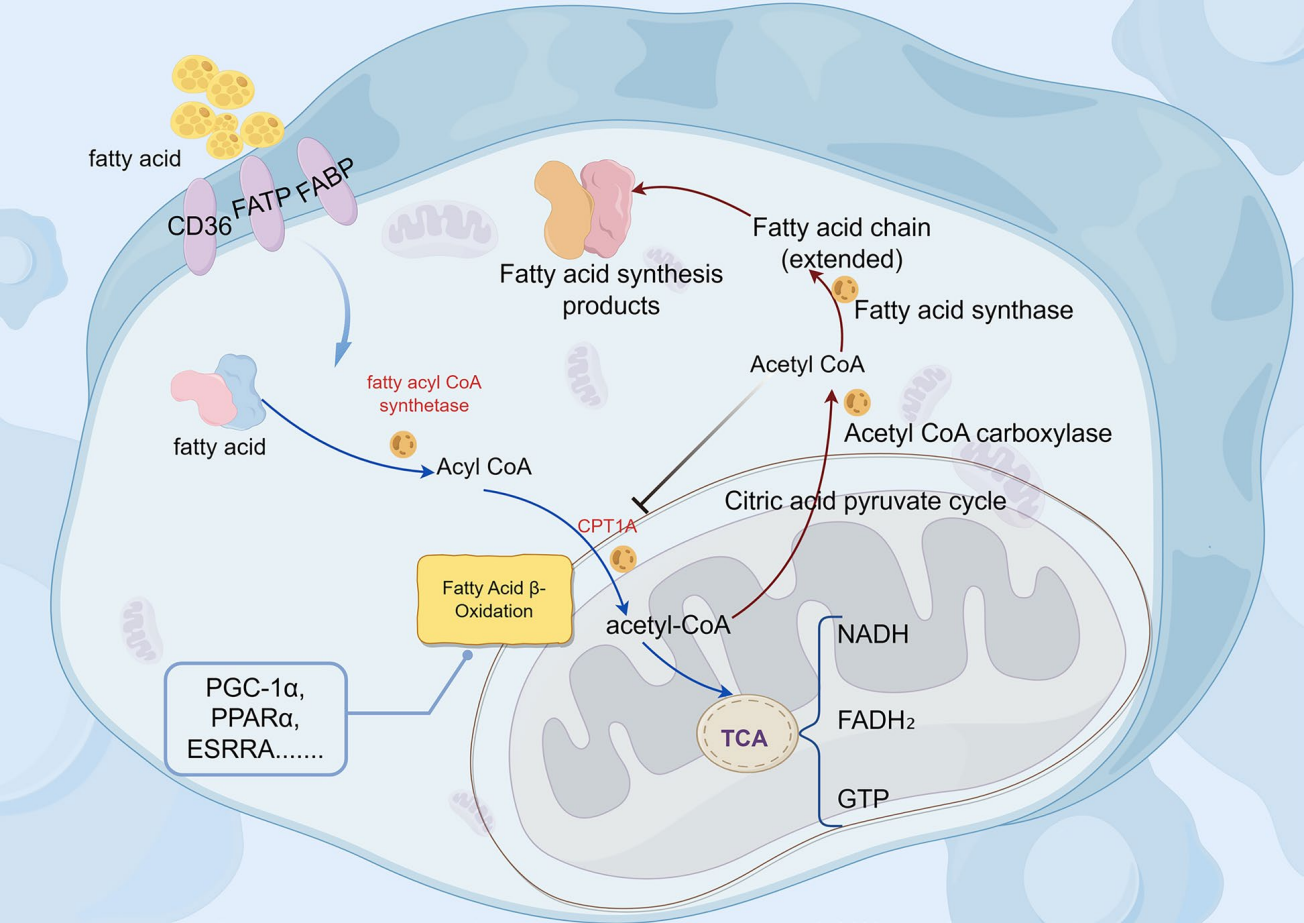
Fatty acid metabolism. The blue arrow represents the fatty acid oxidation pathway, and the red arrow represents the fatty acid synthesis pathway

The kidney is an important organ in the body that regulates metabolic homeostasis. Energy metabolism disorder is a major cause of RF [28], and its energy source depends mainly on mitochondrial fatty acid metabolism [29], which mainly consists of fatty acid oxidation and fatty acid synthesis. Fatty acid oxidation (FAO) mainly occurs in mitochondria or peroxisomes and includes fatty acid esterification, the transfer of acyl coenzyme A (CoA), and β-oxidation of acyl-CoA [30]. When fatty acids are activated into acyl-CoA, they undergo β-oxidation to primarily produce acetyl-CoA, with simultaneous oxidation and dehydrogenation yielding FADH₂ and NADH. Acetyl-CoA then enters the tricarboxylic acid cycle, where it undergoes complete oxidation to generate a small amount of GTP. The resulting NADH and FADH₂ are channeled into the electron transport chain, where they facilitate oxidative phosphorylation to synthesize abundant ATP and release energy. In fatty acid synthesis, acetyl-CoA is transported into the cytosol through the citrate‒pyruvate cycle and then converted into malonyl-CoA by acetyl-CoA carboxylase (ACC) [31]. The newly formed fatty acid chains are continuously elongated by fatty acid synthase, eventually generating products such as palmitic acid (Fig. 1).

In fatty acid metabolism, cluster of differentiation 36 (CD36) and carnitine palmitoyltransferase 1α (CPT1A) regulate the transport of long-chain fatty acids, whereas peroxisome proliferator-activated receptor γ coactivator 1α (PGC-1α), peroxisome proliferator-activated receptors (PPARs) and Estrogen-Related Receptor Alpha (ESRRA) modulate the expression of proteins involved in fatty acid uptake and oxidation, thereby maintaining a dynamic balance among the transport, oxidation, and synthesis of intracellular fatty acids. Studies have shown that impaired β-oxidation of fatty acids and fatty acid overload can damage renal tubular epithelial cells, podocytes, and the renal interstitium and activate apoptotic, inflammatory, mitochondrial, oxidative, and fibrotic factors, leading to the accumulation of ECM and ectopic fat and thereby promoting renal injury and fibrosis [6, 32]. Maintaining the homeostasis of fatty acid metabolism can delay the progression of CKD and has become an important target for improving RF.

### Renal tubules: impaired FAO is a core driver of RF

Renal tubules, especially proximal tubules, have high energy demands and are rich in mitochondria, with FAO being their preferred energy source [33]. Under the influence of multiple factors, FAO is impaired, and metabolic disorders constitute a core driver of RF.

#### Initiation of lipid metabolism-related miRNAs: targeted regulation of core transcription factors

MicroRNAs (miRNAs) are a class of small noncoding RNAs with a length of approximately 22 nucleotides [34]. They regulate gene expression by binding to the mRNAs of target genes and play an important role in RF. Miguel et al. identified specific miRNAs involved in the characteristic metabolic disorders of RF, emphasizing their role in regulating fatty acid oxidation metabolism during RF [34]. A growing body of evidence indicates that specific miRNAs affect the occurrence and development of RF by regulating oxidative metabolism and mitochondrial function.

MiR-21 targets peroxisome proliferator-activated receptor α (PPARα), mitochondrial inner membrane protein (Mpv17L), and reversion-inducing cysteine-rich protein with kazal motifs (RECK), downregulating their expression. This interaction disrupts the lipid metabolism balance and accelerates renal injury and fibrosis [35]. MiR-150 and miR-495 may reduce the FAO-related oxygen consumption rate by inhibiting the expression levels of CPT1A, PGC1α, and mitochondrial transcription factor A (TFAM) and synergizing with TGF-β to promote fibrosis [34].

The loss of expression or functional inhibition of miR-34a and miR-33 can promote FAO, reduce abnormal lipid accumulation in renal tubular cells, improve mitochondrial function, and alleviate RF [36, 37]. In mice with unilateral ureteral obstruction (UUO), miR-9-5p can prevent the downregulation of FAO-related genes, block TGF-β1-induced bioenergetic disorders, induce reprogramming that affects metabolic disorders and mitochondrial dysfunction in renal tubular epithelial cells, and trigger a protective response against chronic kidney injury and RF [38]. MiR-668 can target PPARα, positively regulate the PPARα/PGC-1α pathway, improve fatty acid metabolism and mitochondrial function, exert antioxidative stress and anti-inflammatory effects, and alleviate RF [39].

Exosomes, as natural nanocarriers, can deliver miRNAs to kidney cells in a targeted manner, achieving precise regulation. Exosomal miRNAs not only serve as therapeutic carriers but also act as early diagnostic markers for RF. Studies have shown that miR-181a has diagnostic value in all stages of CKD [40]. The level of urinary exosomal miR-200b decreases significantly with the progression of RF [41]. The regulation of RF by exosomes derived from renal tubular epithelial cells (RTECs) through miRNA signaling networks further reveals the key mechanisms of RF. Currently, studies have confirmed that miR-330-3p in exosomes from renal tubular epithelial cells can inhibit the expression of CREBBP and promote RF [42]. Research on exosomal miRNAs regulating fatty acid metabolism to improve renal fibrosis offers a new perspective that can lead to new directions for the development of exosome-engineered "intelligent drug delivery systems".

#### Transcriptional network: dysregulation of the PPARα/PGC-1α core axis and CPT enzyme-driven mechanisms

Transcription factors such as PPARα, PGC-1α, and ESRRA form a key transcriptional network that regulates mitochondrial biogenesis and FAO. They can regulate lipid metabolism by directly activating target genes such as CPT1A. The loss of function of this axis leads to the inhibition of CPT enzyme expression, triggering lipid accumulation and energy crisis and synergizing with inflammation/epithelial‒mesenchymal transition (EMT) signals to drive RF.

Deficiency of PPARα can increase lipid accumulation and accelerate RF [43]. In renal tubular cells, signal transducer and activator of transcription 6 (STAT6) transcriptionally inhibits the expression of PPARα through the sis-inducible element located in the protein promoter region, thereby increasing lipid accumulation, increasing fibrosis-related proteins, and participating in renal tubulointerstitial fibrosis [44]. Knockout of Krüppel-like factor 14 (KLF14) and Krüppel-like factor 15 (KLF15) in renal tubular cells, as well as activation of activating transcription factor 6α (ATF6α) overexpression, can inhibit PPARα, reduce FAO mitochondrial activity, lead to lipid accumulation and insufficient energy supply in renal tubular cells, increase apoptosis, and ultimately cause lipotoxicity-induced RF [45–47]. Activating PPARα to regulate fatty acid metabolism has a certain therapeutic effect on kidney diseases. For example, the PPARα agonist fenofibrate can alleviate lipotoxicity and ameliorate renal injury and fibrosis by restoring the expression of FAO-related metabolic enzymes [48]. The novel pan-PPAR agonist MHY2013 has also been shown to reduce the degree of RF [49]. Tofogliflozin can increase expression of the β-oxidation genes ACOX1 and CPT1 by upregulating PPARα, thereby reducing renal lipid deposition [50].

PGC-1α is a major transcriptional coactivator of genes involved in mitochondrial synthesis and oxidative metabolism in most cells [51]. Deficiency of lipin-1, a lipid metabolism-related molecule, downregulates FAO by inhibiting PGC-1α/PPARα-mediated CPT1A/hepatic nuclear factor 4 alpha (HNF4α) signaling and upregulates sterol-regulatory element binding protein (SREBP) to promote fat synthesis, thereby causing renal tubular epithelial cell damage and exacerbating fibrosis [52]. Overexpression of Twist-related protein 1 (TWIST1) downregulates the transcription of PGC-1α and further inhibits the expression of FAO-related genes such as PPARα, CPT1, and ACOX1, leading to mitochondrial dysfunction and increased production of profibrotic factors, which results in RF [53]. S100A7a interacts with β-catenin to downregulate the expression of PGC-1α [54]. Specific deletion of S100a7a in renal tubular cells can increase FAO and reduce lipid peroxidation, thereby improving renal function and alleviating RF induced by UUO and unilateral ischemia‒reperfusion injury. The PGC1α activator ZLN005 can mitigate mitochondrial damage and improve renal function [55]. Metformin is an indirect activator of PGC-1α. In renal injury models, metformin can restore the transcriptional level of PGC-1α, regulate mitochondrial viability and dynamics, and alleviate RF [56].

ESRRA is abundantly transcribed in renal tubular cells, and its inhibition leads to impaired FAO and oxidative phosphorylation, as well as changes in the differentiation state of renal tubules, which is closely associated with RF [57]. In addition, Bmal1 is a core circadian rhythm transcription factor. The deficiency of Bmal1 in proximal renal tubules may result in the downregulation of genes related to FAO, weakened ATP production, disrupted renal metabolic homeostasis, exacerbated renal injury, and aggravated RF [58].

In addition to dysregulation of the core axis, other transcription factors can directly promote RF by inducing metabolic reprogramming. Snai1 can induce partial EMT in renal tubular epithelial cells, initiate metabolic reprogramming of renal tubular epithelial cells, inhibit fatty acid metabolism, and maintain inflammation, thereby leading to RF [59, 60]. FOXK1 can upregulate the transcription of glycolysis-related genes, regulate metabolic switching in renal tubular cells, and enhance glycolytic metabolism and fibrotic lesions [28]. C-Myc, a pleiotropic transcription factor, is highly abundant in renal tubular epithelial cells and enhances glycolysis, inhibits FAO, and exacerbates RF [61]. HIF-1 is a heterodimeric nuclear transcription factor that plays an important role in cellular responses to hypoxia [62]. It is expressed in renal tubular cells and exerts a key regulatory effect on the switch from FAO to glycolysis [63]. During fibrosis of renal proximal tubules, increased expression of HIF-1α leads to cellular metabolic reprogramming from FAO to glycolysis along with lipid accumulation [64, 65]. HIF-1 has a dual role in the progression of RF. Studies have shown that after PHD is inhibited with enarodustat, HIF-1 mediates the upregulation of glycogen synthesis genes and exerts a protective effect on the kidney by increasing renal glycogen storage [66]. The upregulation of mitochondrial uncoupling protein 2 (UCP2) leads to tissue hypoxia by activating HIF-1, which in turn stimulates lipid deposition and ECM accumulation, thereby promoting RF [67]. Diosmin can alleviate fibrosis by inhibiting HIF-1α [68].

Carnitine palmitoyltransferase (CPT) is a key rate-limiting enzyme system for FAO to enter mitochondria. CPT is composed of two independent proteins: CPT1, located on the outer mitochondrial membrane, and CPT2, located on the inner mitochondrial membrane. Among them, deficiency of CPT1A, a key executor of the PPARα/PGC-1α axis, directly leads to FAO dysfunction, which is crucial for renal energy metabolism [69]. Studies have shown that treating renal tubular epithelial cells with CPT1A inhibitors can increase the expression of the typical fibrotic genes α-SMA and vimentin [6]. In patients with CKD, the degree of fibrosis is significantly correlated with decreased levels of CPT1A [70, 71]. In a diabetic nephropathy (DN) model, Nε-(carboxymethyl)-lysine (CML), a key component of advanced glycation end products (AGEs), has been confirmed to induce a decrease in CPT2 expression, thereby impairing mitochondrial FAO and ultimately promoting the development of RF [72]. Moreover, overexpression of CPT1A in a mouse model of fibrosis can restore oxidative metabolic capacity and mitochondrial number and function while reducing the dependence on glycolysis and excessive activation of AMPK. It effectively lowers cellular inflammation levels, reduces apoptosis, alleviates transforming growth factor-β (TGF-β)-mediated epithelial cell damage, and improves RF [29]. FOXA1 overexpression can increase the level of CPT1A. Canagliflozin can improve FAO and alleviate ferroptosis in RTECs through the FOXA1-CPT1A axis, thereby exerting a renoprotective effect [73].

#### Lipotoxicity cascade: fibrosis driven by multiple mechanisms

Abnormal fatty acid metabolism not only affects cellular energy metabolism but also triggers inflammatory responses, oxidative stress, mitochondrial dysfunction, and apoptosis through multiple pathways, ultimately promoting the occurrence and development of RF.

Lipid accumulation and inflammation promote each other in the development of RF, forming a complex vicious cycle that can ultimately lead to the progressive destruction of renal structure and function. CD36 is a multifunctional receptor for long-chain fatty acids, oxidized lipids, and advanced glycation end products (AGEs), among others, and its overexpression in renal tubular epithelial cells can lead to lipid-specific accumulation. The activation of CD36 can trigger multiple proinflammatory and profibrotic pathways, such as those involving Toll-like receptors, the NLRP3 inflammasome, PKC-NADPH oxidase, Src family kinases, MAPK, and TGF-β [74]. In addition, overexpression of CD36 inhibits the expression of pyruvate dehydrogenase kinase isozyme 4 (PDK4), leading to mitochondrial dysfunction, reduced ATP production, increased AMP levels, and exacerbation of endoplasmic reticulum stress and apoptosis, thereby significantly promoting renal tubular fibrosis [6]. CD36 is a new potential target for RF [75]. Studies have shown that the 5A peptide can antagonize CD36, exert anti-inflammatory effects and delay RF in the kidney [76]. However, there is currently no marketed drug that specifically targets CD36, and research on drugs targeting CD36 to improve RF has shown potential. KIM-1, a member of the immunoglobulin superfamily, is upregulated in injured proximal tubular cells. It mediates increased uptake of palmitate-bound albumin, induces renal tubular cell death and mitochondrial fragmentation, generates proinflammatory factors by activating the NLRP3 inflammasome, and produces profibrotic factors through the DNA damage response, collectively promoting the progression of RF [77]. Lipid-metabolizing enzymes can also promote inflammasome activation to participate in RF. For example, Recombinant acyl coenzyme A synthetase short chain family member 2 (ACSS2) can regulate de novo lipogenesis, leading to the depletion of reduced nicotinamide adenine dinucleotide phosphate (NADPH) and increased levels of reactive oxygen species (ROS), ultimately resulting in NLRP3-dependent pyroptosis and exacerbating RF [78].

Epigenetic mechanisms regulate gene expression without altering the DNA sequence, and they are crucial for cells to respond adaptively to environmental stimuli (such as injury and metabolites) [79]. In RF, epigenetic changes affect the expression of fatty acid metabolism genes, thereby driving the fibrotic process [2]. For example, β-catenin can inhibit the SUMOylation of LKB1, thereby disrupting fatty acid oxidation in renal tubular cells and triggering RF [80]. Rosiglitazone (RGL), a synthetic agonist of PPARγ (a key factor in fatty acid oxidation), can inhibit the secretion of chemokines and the activation of nuclear factor-κB (NF-κB) in human renal proximal tubular cells (PTCs) by activating PPARγ SUMOylation, thereby suppressing inflammation and improving RF [81]. Deficiency of sirtuin 3 (SIRT3), a mitochondrial NAD+-dependent deacetylase, can lead to excessive acetylation of renal tubular mitochondrial proteins, inhibit the LKB1-AMPK signaling pathway, disrupt fatty acid metabolism, and cause mitochondrial dysfunction, which is an important mechanism underlying the development of RF [82, 83].

Mitochondria are the main organelles for FAO, and accumulated fatty acids can in turn damage mitochondria, with the two influencing each other. The micropeptide regulator of β-oxidation (MOXI) can mediate bidirectional communication between the nucleus and mitochondria during RF. In the early stage of fibrotic stress, MOXI accumulates in mitochondria to promote FAO. However, under long-term stress, MOXI can undergo nuclear translocation and form a transcriptional complex with N-acetyltransferase 14 and c-Jun, thereby promoting the expression of profibrotic genes [84]. Hypoxia induces the overexpression of Hilpda, which leads to triglyceride overload and FAO defects by downregulating adipose triglyceride lipase (ATGL), triggering mitochondrial dysfunction and upregulating profibrotic factors (TGF-β1, α-SMA, type I collagen) while reducing the expression of cyclin-dependent kinase 1 (CDK1) and increasing the CyclinB1/D1 ratio, collectively promoting renal tubular fibrosis [85]. SS-31 is a tetrapeptide that targets AKI. High-fat diet-mediated proximal tubule injury can reduce intracellular lipid accumulation by inhibiting the disruption of mitochondrial function, improving renal function, and preventing RF [86]. The mitochondria-targeted lipophilic antioxidants MitoQ and MitoT protect against renal tubular injury and renal dysfunction by inhibiting mitochondrial damage and oxidative stress, as well as increasing mitochondrial NADPH levels [87].

**Fig. 2:**
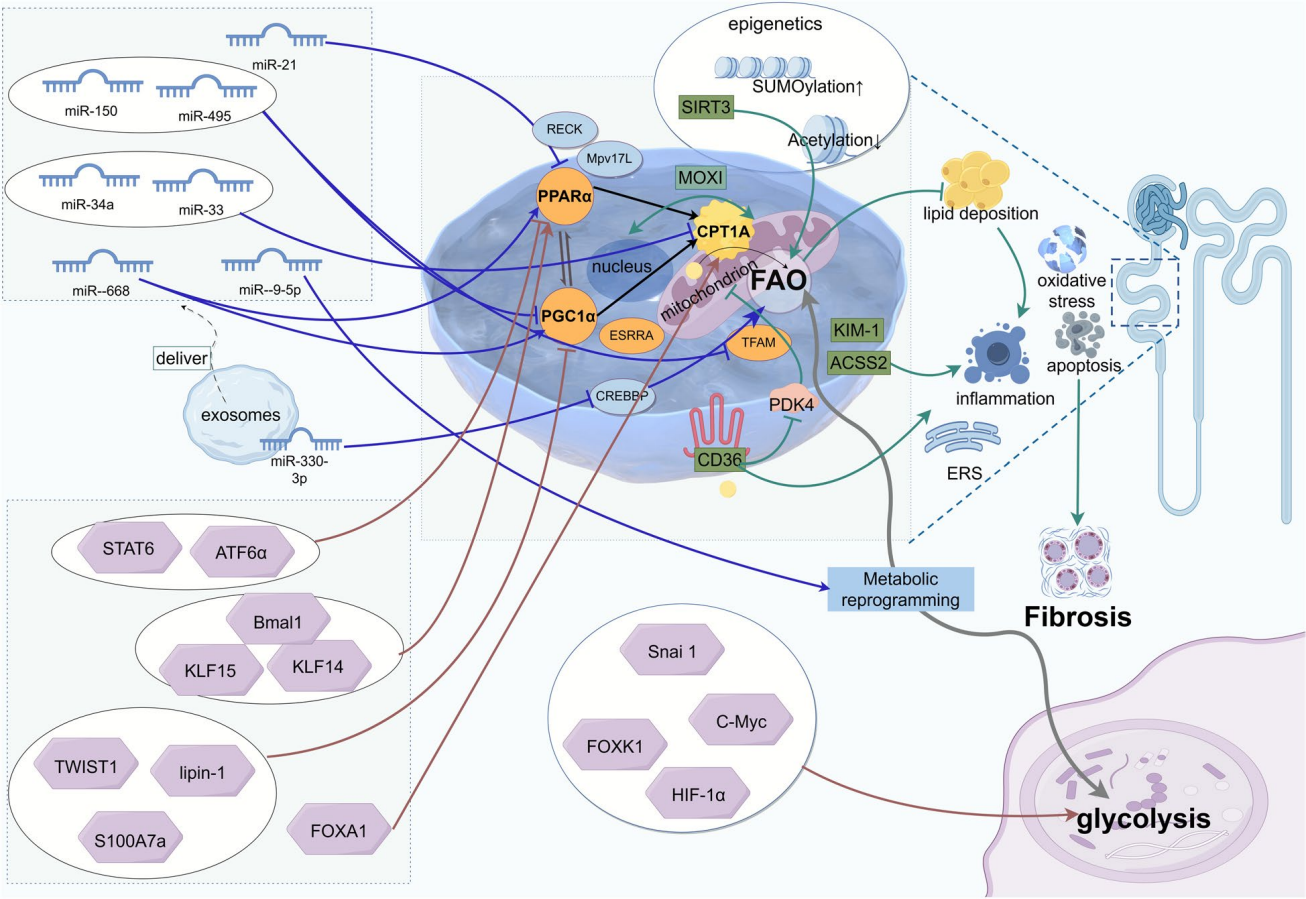
Core mechanisms and regulatory network of RF driven by impaired FAO in renal tubules. The blue line represents miRNA-related driving mechanisms, the red line represents the mechanisms by which some key transcription factors affect FAO, and the green line represents the multimechanistic pathway associated with the lipotoxicity cascade. With the involvement of specific miRNAs, they act on the transcription network centered on the PPARα/PGC-1α axis; inhibit FAO-related pathways; and trigger lipid deposition, oxidative stress, inflammation, metabolic reprogramming, etc. These multiple mechanisms synergistically drive the molecular associations of RF

In summary, with the involvement of specific miRNAs, the dysregulation of the transcription network centered on the PPARα/PGC-1α axis in renal tubules reduces FAO by inhibiting the CPT system, triggering lipid accumulation and mitochondrial damage. Moreover, lipotoxicity mediated by receptors such as CD36 activates inflammasomes. Multiple mechanisms, including metabolic collapse, oxidative stress, and cell death caused by epigenetic reprogramming and hypoxic stress, synergistically drive RF. (Fig. 2).

### Glomerulus: disorders of lipid metabolism damage the filtration barrier

In the glomerular filtration barrier, lipid metabolism disorders mediate mitochondrial oxidative stress and inflammatory responses; induce cytoskeletal rearrangement, autophagy dysregulation, and mitochondrial dysfunction; damage podocytes; disrupt the integrity of the filtration barrier; and ultimately promote glomerulosclerosis and renal interstitial fibrosis (RIF) [88]. Trim63 and recombinant Rho-associated coiled coil containing protein kinase 2 (ROCK2) can inhibit PPARα, aggravate FAO deficiency and mitochondrial dysfunction, trigger lipid deposition, podocyte damage and proteinuria, and induce RF [89, 90]. In glomerular mesangial cells, palmitate increases lipid accumulation through CD36-mediated signaling, activates the transient receptor potential canonical 6/nuclear factor of activated T-cells 2 (TRPC6/NFAT2) pathway, and induces fibrotic responses [91]. Inhibition of Smad3 prevents the loss of synaptopodin and mitochondrial function in palmitate-induced podocytes, thereby alleviating RF [92]. Increased expression of 12/15-lipoxygenase can increase the production of oxidized lipids, promote the expression of proinflammatory genes and TGF-β in mesangial cells, directly induce changes in epigenetic histone modifications involved in the expression of profibrotic genes, and thus promote RF [93].

**Fig. 3:**
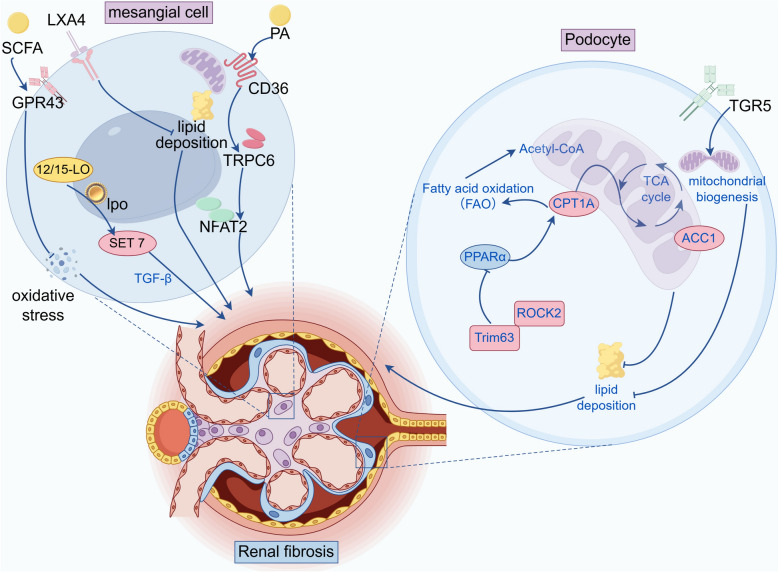
Glomerular filtration barrier injury and RF. In mesangial cells and podocytes, abnormalities in pathways such as Trim63/ROCK2 and CD36/TRPC6/NFAT2 affect lipid metabolism disorders and damage the filtration barrier; protective factors such as SCFAs, TGR5, and LXA4 exert antifibrotic effects by regulating lipid accumulation, mitochondrial function, among others

Protective factors such as short-chain fatty acids (SCFAs), especially butyrate, can inhibit mesangial matrix accumulation. They protect mesangial cells by overexpressing GPR43 to inhibit oxidative stress and NF-κB signaling, induce histone lysine butyrylation to improve glucose and lipid metabolism disorders, and alleviate diabetic nephropathy-induced renal injury and RF [94, 95]. Activation of the Takeda G protein-coupled receptor 5 (TGR5), a G protein-coupled bile acid receptor, can induce mitochondrial biogenesis, prevent renal oxidative stress and lipid accumulation, and reduce podocyte damage and RF [96]. Lipoxin A4 (LXA4) is an endogenous lipid mediator that regulates lipid metabolism, inflammation, and resolution. It can reduce lipid accumulation, inhibit the expression of fibrotic genes in platelet-derived growth factor-induced human renal mesangial cells, and exert anti-RF effects [97] (Fig. 3).

### Renal interstitium: myofibroblast activation and immunometabolism

The core pathological feature of RF is the excessive deposition of collagen-rich ECM in the renal interstitium. Activated myofibroblasts (MFs) are the main cells that produce pathological ECM [98]. Lipid metabolism can affect the activation, proliferation and function of myofibroblasts, thereby influencing the process of fibrosis. Fatty acid binding protein 4 (FABP4), a key mediator of macrophage lipid metabolism and inflammation, can promote the transformation of macrophages into myofibroblasts, facilitate lipid deposition, increase the content of free fatty acids, and accelerate RIF [99]. Calmodulin 2 (CaM2), a key actin filament-related regulatory protein, can independently regulate renal lipid accumulation and FAO pathways by mediating cellular mechanical and chemical signals, thereby promoting RF [100]. Resolvin E1 (RvE1), a metabolite of eicosapentaenoic acid (EPA), is a mediator that promotes lipid metabolism [101]. RvE1 can inhibit fibroblast proliferation both in vivo and in vitro and suppress RIF [102].

In RIF, the main characteristic of fibroblast activation into myofibroblasts includes metabolic reprogramming from oxidative phosphorylation to aerobic glycolysis [103–105]. Accumulating evidence indicates that lipid metabolism reprogramming and specific signaling molecules play crucial roles in the activation and persistence of MF and the production of ECM. The generation of MF may be a significant factor in the necessary downstream effects within the metabolism‒fibrosis axis. WWP2 is an E3 ubiquitin ligase. In the renal interstitium, WWP2 can promote RF by inhibiting PGC-1α transcription and regulating the metabolic reprogramming of MF [5]. WWP2 deficiency may increase the activity of the tricarboxylic acid (TCA) cycle and mitochondrial respiratory metabolism, thereby improving RIF. WWP2 represents a novel regulatory node linking ubiquitination, mitochondrial metabolism, and MF functions. Fatty acid transport protein 2 (FATP2) can regulate the expression of profibrotic cytokines by inducing lipid metabolism reprogramming, such as abnormal fatty acid uptake and FAO defects, triggering fibrotic responses to RIF. Inhibition of FATP2 can improve RF by regulating the secretion of profibrotic cytokines, apoptosis, and endoplasmic reticulum stress [106].

**Fig. 4:**
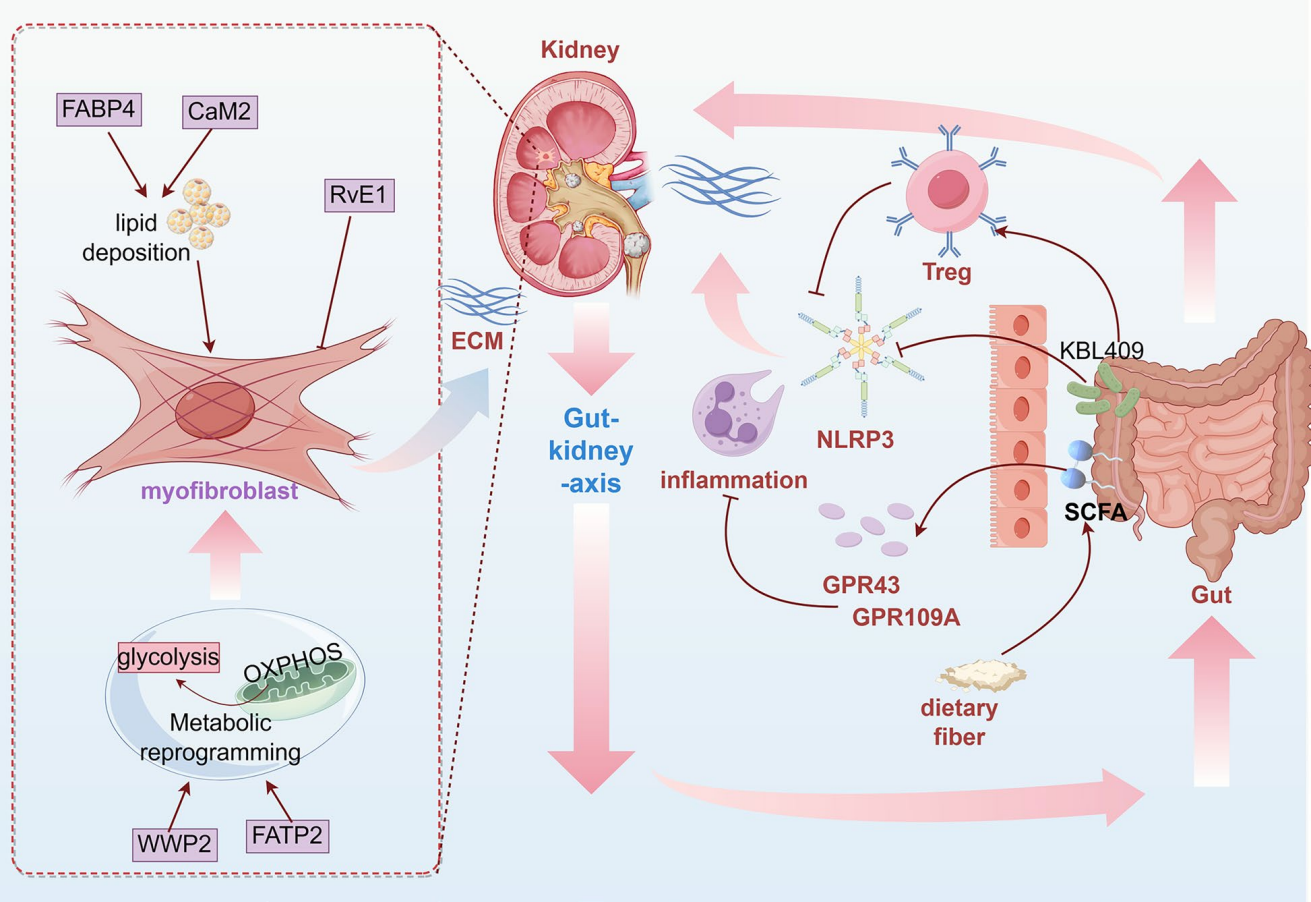
Lipid metabolism modulates myofibroblast activation and immunometabolism in RIF. During RIF progression, MF activation—driven by lipid metabolism dysregulation (FABP4 and CaM2 promote fibrosis, whereas RvE1 suppresses proliferation) and metabolic reprogramming (WWP2/FATP2-mediated glycolysis/oxidative phosphorylation switching)—triggers excessive ECM deposition. Concurrently, gut microbiota-derived dietary fibers and SCFAs activate GPR43/109A receptors, or supplementation with KBL409 modulates immune responses (Treg induction and NLRP3 inflammasome regulation), thereby attenuating renal inflammation and fibrosis

The accumulation of fatty acids can also promote inflammation through the activation of the innate immune system and fibrosis, triggering mitochondrial and renal cell damage [4]. Regulating the intestinal flora to improve fatty acid metabolism can modulate immunity and alleviate RF. Targeting the intestinal flora to improve host fatty acid metabolism is emerging as a new strategy to regulate renal immune responses and mitigate RF. For example, supplementation with *Lactobacillus acidophilus* KBL409 can alleviate adenine-induced intestinal barrier damage in RF mice, reduce macrophage infiltration, increase regulatory T cells, inhibit the NLRP3 inflammasome, and exert immunomodulatory effects to prevent renal injury [107]. In rat fibroblasts and epithelial cells, valproic acid inhibits TGF-β1-stimulated α-SMA expression and induces autophagy, thereby treating RF [108]. Supplementation with dietary fiber can mediate the regulation of immunity and inflammation through the G protein-coupled receptors GPR43 and GPR109A by regulating the intestinal microbiota, enriching SCFA-producing bacteria, and increasing SCFA production, thereby delaying DN and RF [109, 110] (Fig. 4).

### Biological markers

Disorders of fatty acid metabolism constitute an important pathogenic mechanism of RF. The search for relevant noninvasive diagnostic biomarkers is crucial for the early diagnosis, assessment of progression, and development of targeted therapies for RF. Pigment epithelium-derived factor (PEDF) can regulate lipid metabolism by activating the transcription factor PPARα [111]. Clinical cohort studies have shown that the levels of PEDF in serum and urine are positively correlated with the degree of RF, which can serve as a biomarker for RF and has a good evaluative effect on the progression and prognosis of kidney disease in patients with CKD [112]. KIM-1 is involved in lipotoxicity and the inflammatory cascade in the process of RF. In addition, studies have shown that urinary KIM-1 can be used as a biomarker to locate specific segments of damaged tubules, guiding the diagnosis, prognosis and risk stratification of RF [113]. GDF15 can regulate lipid metabolism by activating AMPK-mediated PPARβ [114]. Clinical studies have shown that GDF-15 is associated with an increased risk of renal failure and, as a new type of biomarker, has good predictive performance in terms of risk prediction [115]. RF is correlated with the intestinal microbiota, and SCFAs and other metabolites produced by the intestinal microbiota can affect the physiological functions and pathological processes of the kidney [116]. In patients with CKD, the relative abundance of *Bacteroides fragilis* is significantly reduced, and its abundance is significantly negatively correlated with blood urea nitrogen (BUN) and serum creatinine (SCR), which can serve as important targets for the diagnosis and treatment of RF [117]. The integration of multiomics data will play a key role in biomarker identification, revealing intracellular molecular networks, and elucidating complex disease mechanisms. Combined analysis of proteins and metabolites revealed that lipid metabolism plays an important role in RF and that PPARα may be an important target for RF [118]. In patients with CKD, the expression of PPARα is positively correlated with renal function indicators such as the estimated glomerular filtration rate (eGFR), suggesting that PPARα may play an important role in maintaining renal function and has great potential as a biomarker [119]. The integration of single-cell sequencing and spatial multiomics data further enhances understanding of the metabolic heterogeneity in fibrotic kidneys, providing directions for the development of more precise biomarkers in RF related to fatty acid metabolism. GPX3 can maintain mitochondrial homeostasis and indirectly affect lipid metabolism [120]. Single-cell sequencing and spatial multiomics analyses have revealed that Gpx3, which can serve as an important target for metabolic disorders in kidney diseases, is highly expressed in fibroblasts [121]. Exosomal miRNAs have emerged as novel markers for the diagnosis of RF due to their tissue specificity and stability. Through the integration of single-cell transcriptome sequencing and miRNA omics analysis, miR-330-3p was found to be highly expressed specifically in fibrotic exosomes. Moreover, clinical cohort analysis showed that the level of miR-330-3p in the urinary exosomes of patients with CKD is significantly positively correlated with renal ultrasound elastography parameters, suggesting its potential as a noninvasive biomarker [42].

Currently, evidence regarding potential new biomarkers for evaluating RF remains insufficient, and their large-scale clinical application is not yet adequate. Therefore, further research is needed. By identifying new biomarkers with high sensitivity and specificity, we can overcome the limitations of traditional indicators and achieve early diagnosis, precise classification, and dynamic monitoring of kidney diseases, thereby guiding individualized treatment, delaying disease progression, and improving patient prognosis (Table 1).

**Table 1:** Biochemical markers related to fatty acid metabolism in RF

Biomarker name	Type/source	Mechanism and functional relevance	Clinical significance and diagnostic value	Sample source	References
PEDF (pigment epithelium-derived factor)	Protein	Activates PPARα to regulate lipid metabolism; Positively correlates with renal fibrosis severity	Evaluates CKD progression and prognosis; Serum/urine levels reflect fibrosis severity	Serum, urine	[112]
KIM-1 (kidney injury molecule-1)	Protein	Involved in lipotoxicity and inflammatory cascades	Localizes tubular injury segments; Guides RF diagnosis, prognosis, and risk stratification	Urine	[113]
GDF-15 (growth differentiation factor 15)	Protein	Activates AMPK-mediated PPARβ to regulate lipid metabolism	Significantly positively correlated with renal failure risk; Novel risk prediction biomarker	Serum	[115]
*Bacteroides fragilis*	Gut microbiota	Produces SCFAs that regulate metabolism and immunity; Reduced abundance correlates with renal function decline	Abundance negatively correlates with BUN and serum creatinine (SCr); Potential therapeutic target and diagnostic marker	Feces	[117]
PPARα	Transcription factor	Core regulator of lipid metabolism; Expression positively correlates with renal function	Expression level positively correlates with eGFR; High potential as therapeutic target and prognostic biomarker	Tissue/multiomics	[119]
GPX3 (glutathione peroxidase 3)	Protein	Maintains mitochondrial stability, indirectly regulating lipid metabolism; Highly expressed in fibroblasts	Reflects renal metabolic dysregulation; Potential therapeutic target	Tissue, blood	[121]
miR-330-3p	Exosomal miRNA	Specifically upregulated in exosomes during fibrosis	Urinary exosome levels positively correlate with renal elastography parameters; Noninvasive diagnostic potential	Urinary exosomes	[42]
miR-181a	Exosomal miRNA	Stably expressed across all CKD stages	Holds cross-stage diagnostic value; Novel noninvasive biomarker	Urinary exosomes	[40]
miR-200b	Exosomal miRNA	Expression significantly decreases with fibrosis progression	Fibrosis staging biomarker; Disease monitoring indicator	Urinary exosomes	[41]

In summary, fatty acid metabolism disorders (especially FAO defects) constitute one of the core metabolic bases of RF. They can drive the fibrotic process in renal tubules, glomeruli, and the interstitium through lipotoxicity, mitochondrial damage, inflammatory activation, and epigenetic regulation. Therefore, identifying RF biomarkers related to fatty acid metabolism disorders is crucial for early diagnosis. Targeting the PPARα/PGC-1α-CPT1A axis, lipid receptors (CD36/KIM-1), and inflammasome signaling, along with the intestinal flora and other regulatory targets, represent promising new strategies for delaying RF. Currently, there is still a lack of effective drugs for broad in clinical practice to improve RF by targeting fatty acid metabolism disorders. Moreover, key challenges, such as single-target limitations and the complexity of metabolic regulatory mechanisms, restrict the development and application of new drugs. TCM has unique advantages, including multitarget synergistic intervention, holistic regulation, relatively high safety, and the concept of "preventive treatment of disease", which provides directions for solving these problems. In-depth investigations of Chinese herbal medicines and their bioactive constituents—capable of modulating lipid metabolism, enhancing mitochondrial function, suppressing inflammation, and exerting antifibrotic effects—will elucidate the multi-target synergistic mechanisms underlying their correspondence between TCM pathogenesis and Western pathological processes. Leveraging modern technologies for scientific validation and optimization is poised to advance the development of innovative, more effective, safer therapeutics suitable for long-term use in the prevention and treatment of renal fibrosis, thereby compensating for the limitations of current Western targeted therapies.

## Chinese herbal medicine intervention in fatty acid metabolism to improve RF

### Chinese herbal monomers: targeting key pathways

#### Alkaloids

Alkaloids are commonly found in plants of families such as Menispermaceae, Loganiaceae, Amaryllidaceae, and Solanaceae. A variety of Chinese medicinal herbs with the effects of "clearing damp-heat and resolving stasis-toxin" are rich in alkaloids as their bioactive components, which have been confirmed to exert anti-RF effects by regulating fatty acid metabolism. Capsicum (chili pepper), which is hot in nature and pungent in taste, is specialized in warming and dispelling cold-dampness. Its bioactive component, capsaicin, is a vanillamide alkaloid that can regulate multiple metabolic pathways by activating channels such as AMPK, upregulate TRPV1 expression, downregulate ACC and fatty acid synthase (FAS) levels, improve lipid metabolism disorders [122, 123], and alleviate RF by delaying the activation of MF, inhibiting the TGF-β1-Smad2/3 signaling pathway, suppressing the expression of α-SMA and vimentin, promoting the expression of E-cadherin, and preventing phenotypic changes in renal tubular epithelial cells [124]. Nelumbo nucifera (lotus leaf) can clear heat-dampness and ascend and invigorate yang-qi.Its bioactive component, nuciferine, is a tetracyclic aporphine alkaloid that can effectively reduce renal lipid accumulation by regulating AMPK-mediated expression of FAS and hormone-sensitive triglyceride lipase (HSL) and inhibit inflammation and oxidative stress through the AMPK-mediated Nrf-2/HO-1 and TLR4/MyD88/NF-κB pathways [125]. Peganum harmala exerts the effects of resolving dampness and detoxification. Harmaline, a β-carboline alkaloid that is found in the plant *Peganum harmala*, can inhibit the transcription factor Twist1 and restore FAO by upregulating the expression of PGC-1α, PPARα and their downstream metabolic enzyme CPT1A, thereby alleviating RF [53]. Coptis chinensis is a bitter and cold medicinal herb. Its core component, berberine, a pentacyclic isoquinoline alkaloid, can activate AMPK, increase the expression of PPARα and CPT1, and reverse the defect in fatty acid β-oxidation. Moreover, it can protect podocytes by inhibiting activation of the NLRP3 inflammasome and mitochondrial fission [126, 127]. It interprets the modern pharmacological connotation of "clearing heat and drying dampness".

#### Anthraquinones

Anthraquinones are key bioactive components of purgative and heat-clearing Chinese medicinal herbs such as Rheum palmatum (Rhubarb). Under the guidance of TCM therapeutic principles of "purging fu-organs to eliminate turbid toxin" and "clearing heat to resolve blood stasis", these components can be used to treat syndromes such as excessive heat accumulation and internal stagnation of blood stasis in RF. As a representative bitter-cold purgative medicinal herb, Rhubarb has many bioactive components that exhibit diverse pharmacological effects, such as antioxidant, lipid-regulating, and anti-inflammatory effects [128]. Its core anthraquinone derivative, rhein, can activate the PPARα-CPT1A axis, upregulate sirtuin 1 (SIRT1), block the binding of STAT3 to the Twist1 promoter, promote the expression of CPT1A, and improve lipid metabolism [129, 130]. In a model of chronic allograft nephropathy (CAN), rhein significantly reduced the expression of CTGF and alleviated RF. Emodin can improve dyslipidemia and reduce renal injury by upregulating the levels of GLP-1R and PPAR-γ [131]. Rhubarb enema can increase the level of short-chain fatty acids to improve the intestinal barrier, inhibit intestinal inflammation, and ultimately reduce RF [132].

#### Polyphenols

Polyphenols are a large class of natural organic compounds that are widely found in plant-based foods and are characterized structurally by having multiple phenolic hydroxyl groups. There are various types of polyphenols, including phenolic acids, flavonoids, stilbenes, and lignans, among others. Through the pathways of "clearing heat to nourish yin and resolving turbidity to unblock collaterals", polyphenols exert positive effects on ameliorating lipid metabolism disorders and renal collateral damage caused by internal accumulation of "dampness-turbidity, stasis and heat".

##### Flavonoids

Flavonoids can significantly affect the metabolic process of fatty acids through various mechanisms, thereby exerting a profound effect on the energy balance and lipid homeostasis. Epimedium, which is specialized in tonifying kidney-yang and strengthening muscles and bones. Icariin II, a flavonol glycoside that is mainly present in *Epimedium*, can restore the expression of PPARα in renal tubules; reduce the expression of fibrotic markers such as fibronectin, collagen I, and α-SMA; and improve FAO, mitochondrial function, and RF [133]. This reflects its scientific connotation of "warming and tonifying kidney-yang to assist qi transformation", thereby restoring renal metabolic dynamics and preventing the formation of "turbidity and stasis". Scutellaria baicalensis, a bitter and cold medicinal herb, is a key medicine for clearing heat and drying dampness, purging fire and detoxifying. Its main component, baicalin, may exert antifibrotic effects by increasing the expression of CPT1A, increasing FAO, and inhibiting the TGF-β/Smad signaling pathway [134, 135]. Fisetin is a flavonoid component that is widely present in plant medicinal materials such as *Cotinus coggygria* and *Toxicodendron vernicifluum*. As a CD36 inhibitor, it can reduce the production of ROS, AGEs, and inflammatory cytokines, thereby improving glomerular function and exerting antifibrotic effects [136]. Tartary buckwheat exerts the effects of invigorating the spleen and resolving dampness. Its main component, tartary buckwheat flavonoids (TBFs), can activate the AMPK/ACC pathway and inhibit SREBP-1 to regulate blood lipid levels, reduce lipid deposition in the kidneys, and alleviate renal inflammation and RF [137]. Juglanin, a flavonol compound, improves the expression levels of genes related to FAO, such as CPT1A and PPARα, and reduces the expression levels of genes involved in fatty acid synthesis, such as SREBP-1 and FXR, by inhibiting the nuclear translocation of NF-κB and histone deacetylase 3 (HDAC 3). Additionally, it inhibits inflammation and lipid accumulation to prevent renal injury [138]. Silybum marianum can clear heat and detoxify. The active ingredient of *Silybum marianum* is silymarin, which is a flavonoid. It significantly reduces renal lipid accumulation by upregulating the expression of CPT enzymes, activates FAO, improves oxidative stress, and protects against mitochondrial dysfunction in the kidneys, thereby alleviating renal injury [139]. It can be used to relieve renal injury caused by "internal stagnation of dampness-turbidity and stagnant heat".

##### Nonflavonoid polyphenols

Honokiol, which is present mainly in *Magnolia officinalis*, acts as a SIRT3 activator. It can activate SIRT3 to inhibit the TGF-β1/Smad signaling pathway, thereby reducing the activation and migration of MF, promoting mitochondrial fusion, regulating fatty acid metabolism, and improving UUO-induced RF. Additionally, it can activate AMPK/PGC-1α/CPT1B-mediated FAO to alleviate RF in DN [140, 141]. This reflects the scientific connotation of its efficacy in "drying dampness and promoting qi movement" to restore the transportation and transformation function of the middle jiao, thereby ameliorating metabolic stagnation throughout the body, including the kidneys. Resveratrol (RSV) is a polyphenolic compound derived from various plants, such as rhubarb, grapes, and peanuts. RSV can reduce lipid deposition in the kidneys and ameliorate diabetic renal injury by regulating the JAML/Sirt1 lipid synthesis pathway [142]. It intervenes in the core links of metabolic kidney disease from the perspectives of "activating blood circulation to unblock collaterals" and "clearing heat".

#### Terpenoids

Terpenoids are a class of natural products with diverse structures that are widely present in plants and have significant regulatory effects on fatty acid metabolism. They play a key role in ameliorating the "deficiency in origin and excess in superficiality" pathogenesis associated with RF. Ginseng possesses the effects of strongly tonifying primordial qi, restoring pulse and resuscitating collapse, tonifying the spleen and benefiting the lung. Ginsenoside Rg1 is a triterpenoid compound that is widely present in ginseng. It can inhibit the expression of CD36 and phosphorylated phospholipase C (p-PLC), improve abnormal lipid metabolism in T2DM mice, reduce excessive free fatty acid (FFA) uptake by renal cells, and exert anti-inflammatory and antifibrotic effects [143]. Cornus officinalis has the effects of tonifying the kidney and astringing essence, which can reduce the leakage of refined substances and alleviate the production of turbid pathogens. Morroniside, a major iridoid glycoside and the main component of *Cornus officinalis* can upregulate the expression levels of its downstream target genes, such as ApoE, by activating the PGC-1α pathway, thereby reducing cholesterol accumulation in TECs [144]. Tripterygium wilfordii exerts the effects of dispelling wind and dampness, activating blood circulation to unblock collaterals. Triptolide, a diterpenoid compound that is mainly present in *Tripterygium wilfordii*, improves lipid metabolism disorders and protects renal function by inhibiting inflammation and oxidative stress, regulating dyslipidemia, and reducing lipid deposition in the kidneys [145]. Liquorice can tonify the spleen and replenish qi. The 18α-Glycyrrhetinic acid, a major pentacyclic triterpenoid in liquorice extract, can increase the phosphorylation level of ACCα and upregulate the expression of FAO-related genes such as PPARα and UCP2, thereby exerting renoprotective effects by reducing oxidative stress, lipid deposition, and inflammatory responses [146].

#### Sulfocompounds

**Fig. 5:**
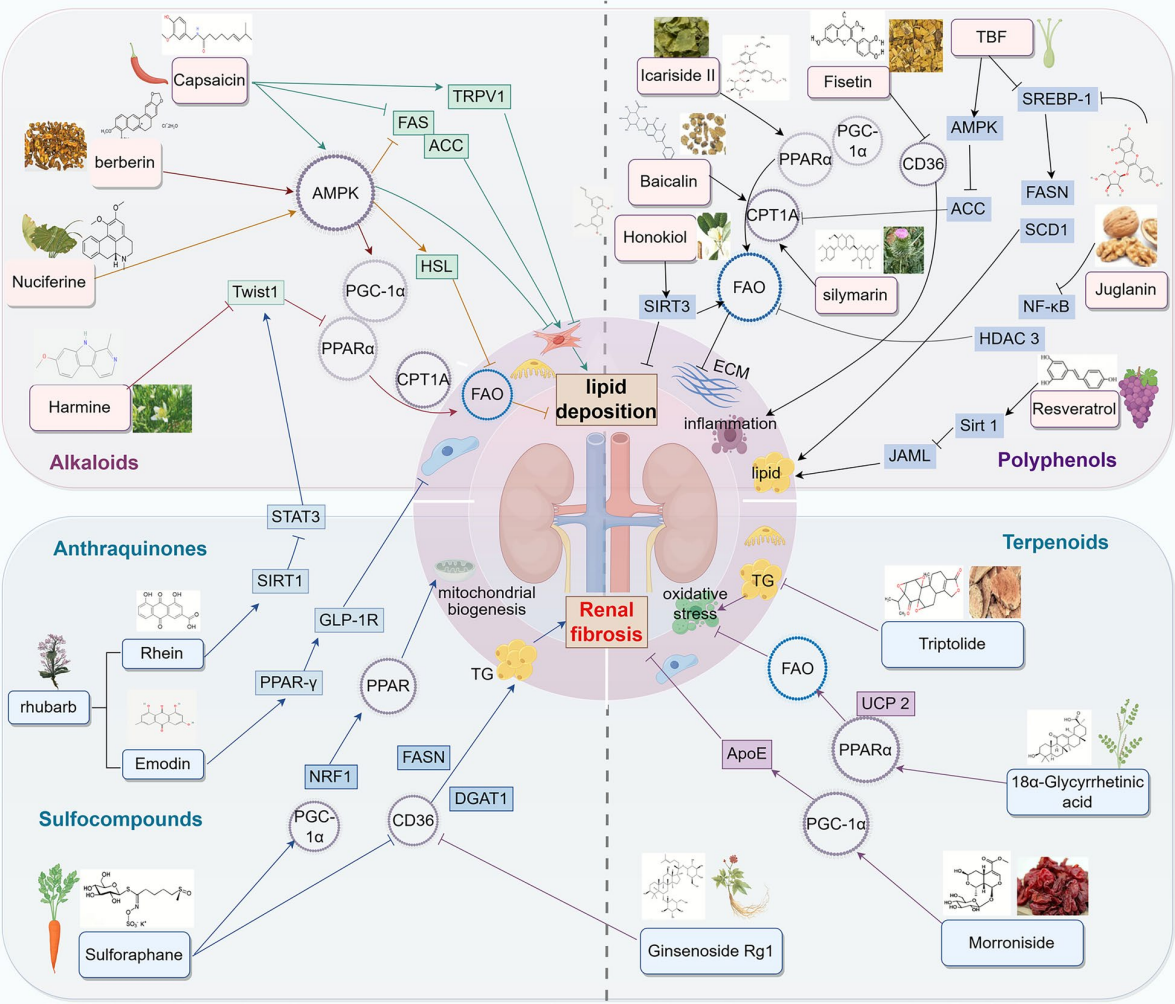
Regulation of fatty acid metabolism by herbal monomers to improve RF-related targets. Alkaloids, anthraquinones, polyphenols, terpenoids, and sulfocompounds in traditional Chinese medicine directly regulate multiple pathways and targets related to fatty acid metabolism and reduce lipid deposition, inflammation, and oxidative stress, thereby exerting anti-RF effects

Sulforaphane is an isothiocyanate compound, which can enhance PGC-1α and nuclear respiratory factor 1 to promote mitochondrial biogenesis; downregulate CD36, sterol regulatory element-binding protein 1, FAS, and diacylglycerol O-acyltransferase 1; increase CPT1A to reduce fatty acid uptake and improve lipid metabolism; downregulate the fission protein dynamin-related protein-1 to reduce mitochondrial fission; restore autophagic flux; improve mitochondrial dynamics; and alleviate RF [147]. The ability of sulforaphane to alleviate lipid accumulation and regulate mitochondrial dynamics for RF improvement reflects the TCM therapeutic principle of eliminating accumulated turbid toxin from the body, regulating qi movement to restore bodily balance, and thereby ameliorating RF (Fig. 5).

### Single Chinese medicine

Some single herbs can improve RF by synergistically regulating fatty acid metabolism through multiple components to resolve turbidity and lower lipids. *Cordyceps sinensis* can tonify the lung and kidney. Modern studies have shown that it can activate the expression of PPARα, ACOX1, and stearoyl-CoA desaturase; inhibit FAS; and regulate the PPARα pathway to reduce renal triglyceride accumulation in diabetic rats, improve renal function, and alleviate RF [148]. Lindera aggregata can promote qi movement, warm the kidney, unblock qi movement in the lower jiao, and eliminate turbid pathogens. Modern studies have demonstrated that it can regulate fatty acid-related metabolites and improve RF by reducing the content of indoxyl sulfate to inhibit the TGF-β/Smad signaling pathway [149]. At the metabolic level, this interprets its mechanism of action in "tonifying the kidney and benefiting essence" to assist qi transformation, eliminating internal stagnation of "phlegm-dampness", thereby improving renal function and alleviating RF. Myrrh is specialized in activating blood circulation to relieve pain, reducing swelling and promoting granulation. Modern pharmacological studies have shown that myrrh essential oil can reduce the levels of renal injury markers such as KIM-1 and exert renoprotective effects through antioxidation, anti-inflammation, and anti-apoptosis [150]. Its bioactive component, Z-Guggulsterone (Z-GS), can inhibit the expression of α-SMA, TGF-β, and collagen Ⅳ to alleviate RF in mice [151]. The ethanol extract of Commiphora myrrha (Nees) Engl. can alleviate steatosis and renal lumen stasis, and reduce hepatorenal injury in diabetic rats [152]. Rhizoma Chuanxiong is also a typical representative of blood-activating medicinal herbs. Network pharmacology studies have indicated that it can regulate lipid metabolism-related pathways to treat diabetic nephropathy [153]. Poria cocos can invigorate the spleen and resolve dampness; it can treat RF by promoting the transportation and transformation of water-dampness and metabolizing turbid pathogens. Modern research has shown that it can improve CKD by regulating fatty acid metabolic pathways [154].

The above representative medicinal herbs for replenishing qi, activating blood circulation, and clearing turbid toxin reflect the organic combination of TCM therapeutic principles targeting the core pathogenesis of "deficiency in origin and excess in superficiality" in RF and Western medicine’s targeted therapy for fatty acid metabolism, which to a certain extent clarifies the scientific connotation of Chinese medicines in improving RF.

### Chinese herbal compounds: synergistic multitarget regulation

**Fig. 6:**
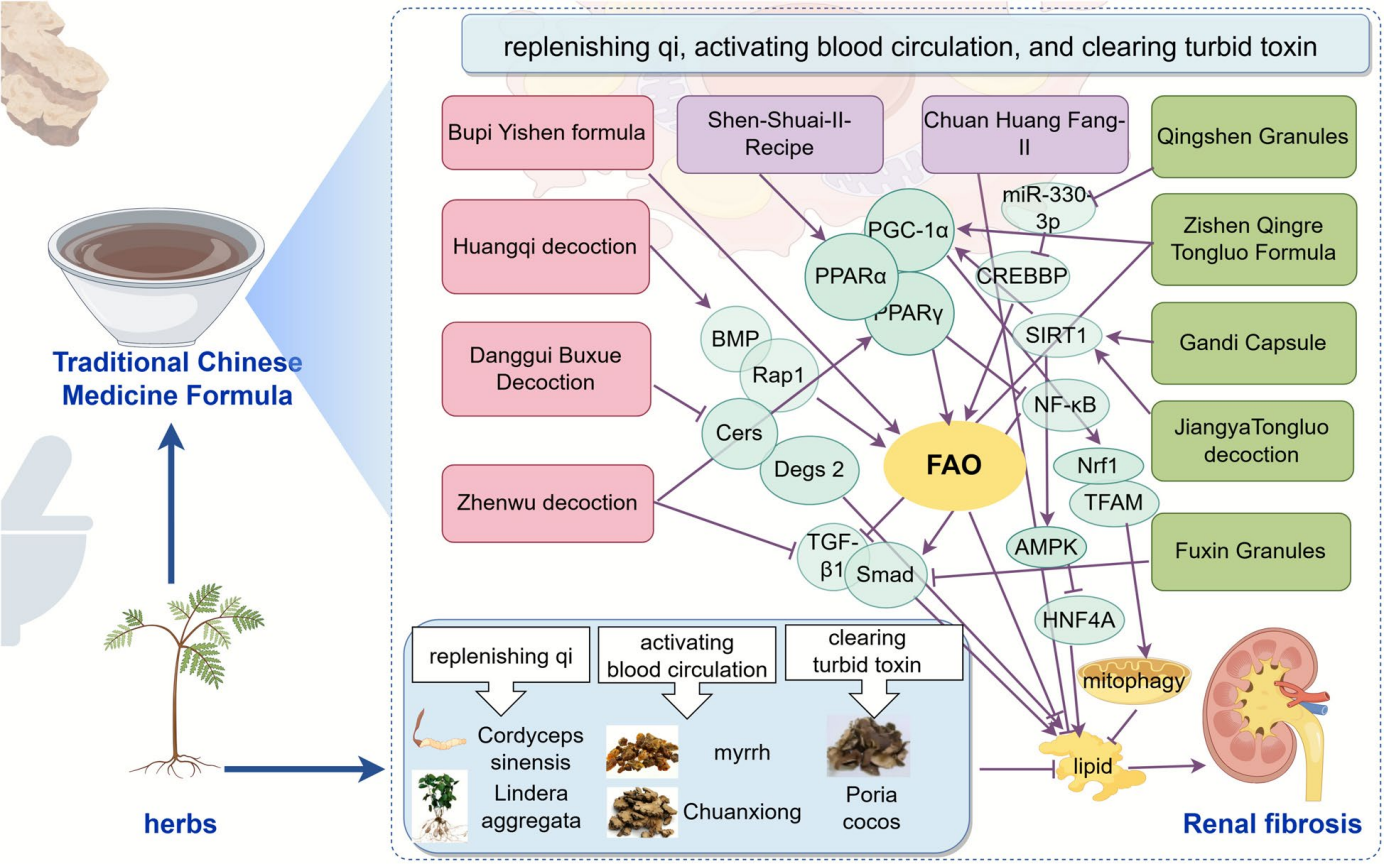
Chinese herbal compounds regulate fatty acid metabolism and improve RF-related targets. Through the compatibility of Chinese herbal compounds, multiple signaling pathways, such as the SIRT1, PGC-PPAR family, NF-κB, and TGF-β1/Smad pathways, are regulated, and processes, including FAO, lipid metabolism, and mitophagy, are affected, thereby achieving synergistic anti-RF effects through multiple pathways

In clinical practice, the intricate interweaving of syndromes and pathological networks often exceeds the regulatory capacity of single herbs. Under the guidance of the "monarch, minister, assistant and guide" compatibility principle and the concept of treatment based on syndrome differentiation, Chinese herbal compounds achieve multi-pathway synergistic intervention, exert systematic regulation on the complex "deficiency in origin and excess in superficiality" pathogenesis of RF, and restore bodily metabolic homeostasis. Bupi Yishen Formula targets the root cause of spleen-kidney deficiency, with the therapeutic efficacy of invigorating the spleen and replenishing qi, tonifying the kidney to consolidate the root. In a multicenter clinical trial, Bupi Yishen Formula was confirmed to have a better effect on protecting renal function than losartan. Mechanistic studies have shown that it exerts a protective effect on RF by restoring FAO in renal tissues, reversing defective fatty acid oxidation and intracellular lipid accumulation, inhibiting renal tubular apoptosis, and improving renal function [155]. Danggui Buxue decoction can reduce the content of ceramides (Cers), phosphatidylethanolamine, and phosphatidylcholine in the kidneys of mice by downregulating the transcription levels of Degs2 and Cers, reducing lipid accumulation, promoting Akt phosphorylation, and alleviating lipid accumulation as well as renal injury [156]. Huangqi decoction can regulate lipid metabolism and improve the progression of DN by increasing the protein expression of BMP2, BMP7, and BMPR2 and increasing the activity of Rap1 while inhibiting Smad1 and phosphorylated ERK and promoting the binding of Rap1 to GTP [157]. Zhenwu decoction, which functions to warm yang and induce diuresis, can reduce the expression level of TGF-β1, upregulate the expression level of PPARγ, increase energy metabolism, alleviate oxidative stress, and regulate fibrotic cytokines, thereby exerting renoprotective effects and improving RF [158]. Qingshen Granules possess the effects of clearing heat, resolving stasis and dispelling dampness; clinical studies have demonstrated its significant efficacy in treating CKD patients with damp-heat syndrome [159]. Further mechanistic studies indicate that it can regulate glucose and lipid metabolism, and ameliorate RF by modulating exosomes, reducing miR-330-3p levels and increasing CREBBP expression [160, 161]. Zishen Qingre Tongluo Formula exerts renal protective effects by nourishing kidney yin, clearing damp-heat and unblocking renal collaterals to improve RF. Mechanistic studies have shown that it can upregulate PGC-1α, PPARγm and PPARα; downregulate fibronectin, collagen Im and Smad3; promote renal FAO through the TGF-β1/Smad3 signaling pathway; and improve RF [162]. Gandi Capsules have the effects of nourishing yin, activating blood circulation, replenishing qi and unblocking collaterals, and can be used for treating diabetic nephropathy. Specific mechanistic studies suggest that it may regulate podocyte lipid metabolism through the SIRT1/AMPK/HNF4A pathway and inhibit lipid accumulation in the kidneys [163]. Jiangya Tongluo decoction can calm the liver and subdue yang, activate blood circulation and unblock collaterals, and eliminate stagnant turbid toxins from the body. Modern studies have shown that it increases the expression of SIRT1, PGC-1α, Nrf1, and TFAM, activates PINK1/Parkin-mediated mitophagy; and regulates the SIRT1/PGC-1α-mitophagy pathway, clears damaged mitochondria, and thereby exerts renoprotective and antifibrotic effects in hypertensive nephropathy [164]. Fuxin granules can reduce the number of renal lipid droplets, improve lipid deposition and inflammation, and alleviate fibrosis by regulating the TGF-β1/Smad and VEGF/VEGFR2 signaling pathways [165]. Shenshuai II Formula has the effects of replenishing qi and resolving stasis, which is of great significance for improving RF. It can inhibit the PPARα/NF-κB/NLRP3 pathway by promoting PPARα-mediated FAO, thereby reducing renal tubular inflammation, weakening fibroblast activation, and improving RF [166]. Chuan Huang Fang-II, composed of Rhizoma Chuanxiong and Rhubarb, has the effects of detoxifying and resolving stasis. It has been clinically confirmed to improve renal function and regulate blood lipid levels in patients; further mechanistic studies have indicated that it exerts therapeutic effects by regulating lipid metabolism [167] (Fig. 6).

### Gut–kidney axis

According to TCM theory, the spleen and kidney are closely correlated, and the intestine acts as a hub connecting them. Impaired intestinal function may impair qi-blood circulation and functional coordination between the spleen and kidney; accordingly, the gut-kidney axis plays a vital role in the pathogenesis of RF. The intestine and kidney interact with one another in terms of fatty acid metabolism. SCFAs produced by the intestine with the participation of the intestinal flora can be absorbed into the blood and participate in renal fatty acid metabolism. Some Chinese herbal medicines can regulate the intestinal flora, improve fatty acid metabolism, and alleviate RF. Curcumin can increase the relative abundance of SCFA-producing bacteria such as Lactobacillus and Ruminococcaceae [168]. Ootheca Mantidis can increase the contents of *Akkermansia muciniphila* and glutamine, regulate the intestinal microbiota, strengthen the intestinal barrier, improve fatty acid metabolism, and exert antioxidant, antiapoptotic and anti-inflammatory effects to reduce renal tubulointerstitial fibrosis and improve renal function [169]. Paotianxiong is a processed product of Radix Aconiti Lateralis Preparata, which exerts effects of warming and tonifying the spleen and kidney as well as replenishing fire to assist yang. Its main component, Paotianxiong polysaccharide, can increase the abundance of Lactobacillus murinus and Bacteroides fragilis, regulate dysregulated intestinal flora, and improve renal function in CKD rats [170]. As a classic herb pair that replenishes qi and activates blood circulation, *Astragalus membranaceus*-*Salvia miltiorrhiza* plays an important role in regulating lipid metabolism and improving RF, which embodies the TCM therapeutic principle of "replenishing qi to promote blood circulation and activating blood circulation to unblock renal collaterals". *Astragalus membranaceus*-*Salvia miltiorrhiza* can reverse disorders of the flora, increase the number of probiotics that produce butyrate and acetate, regulate butyrate metabolism, elevate the levels of key lipid metabolism regulators such as PCK1 and ACOX1, and alleviate RF caused by calcineurin inhibitors [171]. Further integrated analyses of serum metabolomics and renal proteomics revealed that *Astragalus membranaceus*-*Salvia miltiorrhiza* can regulate the differential metabolites alpha‑Dimorphecolic acid and 12,13‑EPOME, as well as differential proteins including Lp, Ppara, and Pck1, thereby modulating lipid metabolism and alleviating calcineurin inhibitor‑induced RF [118]. Astra*galus membranaceus-Salvia miltiorrhiza* can also be driven by *Akkermansia muciniphila* and *Lactobacillus murinus,* regulates target metabolites such as 1‑acyl‑sn‑glycero‑3‑phosphocholine and phospho1, modulates pathways related to glucose and lipid metabolism, and thereby ameliorates diabetic nephropathy through the gut‒kidney axis [172]. In hypertensive nephropathy, *Astragalus membranaceus-Salvia miltiorrhiza* can increase the abundance of probiotics such as *Akkermansia muciniphila* and *Lactobacillus intestinalis*, which are related to the production of butyrate and lactic acid as well as the regulation of inflammation. It reduces the levels of metabolites including leukotriene B4 and prostaglandin E2, alters the serum metabolic profile, modulates lipid metabolism-related pathways, and thereby alleviates hypertension-induced renal injury [173]. Astragaloside IV, the active ingredient of *Astragalus membranaceus is found to operate through a gut–transcriptome–metabolome co‑expression network*. It increases the abundance of gut bacteria such as Lactobacillus reuteri and Blautia glucerasea, regulates the responses of target genes (e.g., Ugt1a5, Slc7a11, Pck1) and metabolites (e.g., 3‑Carboxy‑1‑hydroxypropylthiamine diphosphate, UDP‑D‑galacturonic acid, Isopentenyl pyrophosphate), and modulates core networks of energy and substance metabolism, such as the citrate cycle and glycolysis/gluconeogenesis, inhibit uremic toxins, and improve RF [174]. Salvianolic acid C, the active ingredient of *Salvia miltiorrhiza*, upregulates PGC‑1α, reduces the lactic acid concentration in renal tissues or cells, increases PCK1 expression, and inhibit RF by regulating renal gluconeogenesis mediated by glucose and lipid metabolism [175]. Huangqi Danshen decoction can upregulate stearoyl-CoA desaturase 1 (SCD 1) and inhibit cGAS/STING signaling to regulate lipid metabolism, thereby improving RF [176].

**Fig. 7:**
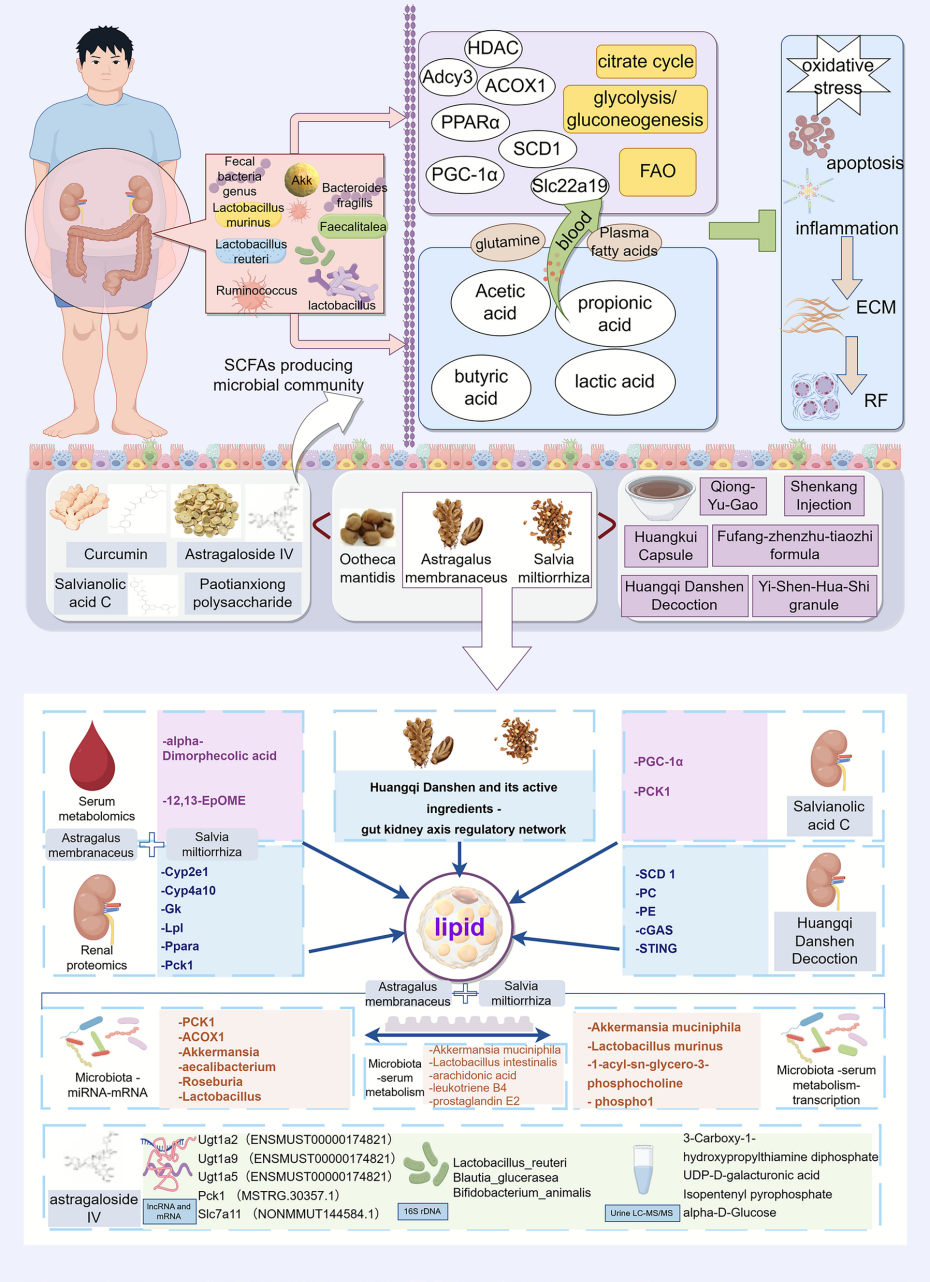
Chinese herbs regulate fatty acid metabolism via the gut–kidney axis to ameliorate RF. Chinese herbs and their different combinations can regulate the intestinal flora and produce SCFAs, thereby affecting pathways and targets related to fatty acid metabolism and ultimately influencing the progression of RF. Among them, *Astragalus membranaceus-Salvia miltiorrhiza* is a key core herb pair that systematically regulates networks such as transcription and metabolism in the blood, urine, kidneys, and intestines and affects the progression of RF through multiple mechanisms

Yishen Huashi granules exert effects of invigorating the spleen and tonifying the kidney, resolving dampness and eliminating turbidity to restore the transportation and transformation function of the middle jiao, so that refined substances can be distributed and dampness-turbidity eliminated, thereby treating RF. Under the guidance of the spleen-kidney correlation theory, exploration into the modern mechanisms underlying Yishen Huashi granules in treating RF has revealed that it can increase the intestinal flora content, such as Lactobacillus and *Lactobacillus murinus*, as well as serum metabolites such as phosphatidylethanolamine through the "gut‒kidney axis", thereby regulating the expression of mRNAs such as Adcy3 in the kidney and adjusting pathways related to glucose and lipid metabolism to improve DN [177]. Huangkui capsules mainly composed of damp-heat-clearing medicinal herbs, can increase Faecalitalea and Muribaculum, restore plasma fatty acid levels, improve the levels of circulating metabolites, and alleviate RF [178]. Fufang Zhenzhu Tiaozhi Formula can alleviate the intestinal flora imbalance, increase the content of SCFAs (including propionate and butyrate) and the level of the SCFA transporter Slc22a19 in the kidney, downregulate the expression of kidney genes related to inflammation and fibrosis, and ameliorate renal injury [179]. Qiongyu Ointment can regulate the intestinal flora; increase the production of SCFAs such as acetate and butyrate; inhibit the expression and activity of histone deacetylases; and reduce the accumulation of uremic toxins, thereby alleviating fibrosis, inflammation, and renal tissue apoptosis [180]. Shenkang injection can regulate fatty acid metabolism to reduce the expression of acetate, restore serum fatty acid metabolites, negatively regulate the TGF-β/Smad signaling pathway, and inhibit EMT [181] (Fig. 7).

In summary, under the guidance of core TCM therapeutic principles of replenishing qi, activating blood circulation, and clearing turbid toxin, Chinese herbs and their active ingredients form a multidimensional anti-RF system through various approaches, such as monomer-targeted pathways, multicomponent synergy of single herbs, multitarget regulation of compound prescriptions, and intestinal flora modulation. These approaches synergize with inflammatory, oxidative stress, and fibrotic signaling pathways related to fatty acid metabolism, providing abundant ideas for the prevention and treatment of RF. Linking TCM pathogenesis and therapeutic principles to modern pharmacological mechanisms and targets embodies the modern scientific connotation of TCM holism and treatment based on syndrome differentiation, providing abundant novel insights for the prevention and treatment of RF.

## External traditional Chinese medicine therapies can improve RF by interfering with fatty acid metabolism

Under the guidance of TCM theories of treating internal and external disorders simultaneously and correlation between meridians, collaterals, zang-fu organs, TCM external therapies (such as acupoint application, auricular point therapy, acupuncture, moxibustion, and Chinese herbal enema) intervene in the treatment of RF through a "non-oral" approach. They can provide a low-toxicity potential solution for improving RF by restoring the homeostasis of fatty acid metabolism. They also play important roles in enhancing treatment efficacy, reducing toxicity, improving symptoms, and increasing patients' quality of life. In clinical practice, moxibustion at acupoints such as Shenshu (BL 23), Pishu (BL 20), Guanyuan (CV 4), and Zusanli (ST 36) warms and tonifies spleen-kidney yang, promotes qi transformation and diuresis, which reduces blood lipid levels in patients with kidney disease and improve clinical symptoms and renal function through a "non-oral" intervention approach [182]. Acupuncture at acupoints such as Quchi (LI 11), Zhigou (SJ 6), Hegu (LI 4), and Xuehai (SP 10) alleviates clinical symptoms, regulates glucose and lipid metabolism, and mitigates renal damage in patients [183]. This embodies the mechanism of "unblocking meridians and collaterals" to modulate zang-fu metabolic function. Additionally, studies have shown that different combinations of acupoints have varying effects on reducing blood lipid levels, among which the combination of the Quchi (LI 11), Zhongwan (CV 12), and Fenglong (ST 40) acupoints has better effects on lipid metabolism [184]. As mentioned earlier, GDF-15 is an important potential biomarker for RF; combined therapy of acupuncture and oral Chinese medicine can reduce GDF-15 levels and improve renal function in patients with early diabetic nephropathy [185]. In dialysis patients, acupuncture treatment can help preserve residual renal function and improve insomnia and depression [186–188]. Acupuncture can also alleviate RIF by inhibiting the TGF-β/Smad pathway [189]. In aged renal tissues, electroacupuncture can inhibit the excessive expression of IL-1β and α-SMA and exert anti-RF effects by inhibiting the PI3K/Akt pathway [190]. Another in vivo study revealed that electroacupuncture at acupoints such as Guanyuan (CV 4), Zusanli (ST 36), Zhongwan (CV 12), and Fenglong (ST 40) can upregulate the expression of the fatty acid metabolism-related transcription factors FoxO1 and PGC-1α in rat mesangial cells, improve renal function, and protect the kidneys [191]. Moreover, electroacupuncture at the Guanyuan (CV 4) and Zhongwan (CV 12) acupoints, as well as the bilateral Zusanli (ST 36) and Fenglong (ST 40) acupoints, can regulate blood lipid levels in rats, reduce triglycerides (TG) and total cholesterol (TC), and alleviate podocyte damage [192].

Chinese herbal enema and colon dialysis act directly on the intestine, which is a direct embodiment of the TCM therapeutic principle of "unblocking the fu-viscera and descending turbidity". Clinical evidence shows that colon dialysis combined with Chinese herbal enema can treat chronic renal failure by reducing the serum creatinine level and delaying the progression of kidney disease [193]. High-position colon dialysis combined with retention enema of Chinese herbs can reduce patients' blood lipid levels and decrease the accumulation of uremic toxins [194]. Chinese herbal enema can also regulate the composition and abundance of intestinal microorganisms, thereby improving the progression of CKD [195]. In clinical practice, Chinese herbal enema is often used in combination with Western medicine for treatment; for example, the combination of Chinese herbal medicine and allopurinol can significantly reduce patients' serum uric acid levels, improve renal function and lipid metabolism, and alleviate clinical symptoms [196].

Acupoint application is based on the theory of meridians in traditional Chinese medicine. It improves renal function and delays renal fibrosis through the "medicine–acupoint–meridian" pathway. It features a simple operation and few adverse reactions, and it can serve as an important external treatment method in the comprehensive management of kidney diseases. A study based on ultrahigh-performance liquid chromatography‒tandem mass spectrometry revealed that the time for maintaining effective blood drug concentrations through transdermal patch therapy at acupoints is significantly longer than that of nonacupoint administration, which is conducive to the long-term and stable exertion of drug effects [197]. Studies on kidney diseases have demonstrated that applying drugs at the Back-Shu Points and Front-Mu Points acupoints can effectively reduce uric acid levels in rats, upregulate the protein expression of renal OAT1 and ABCG2, and alleviate renal damage [198]. Auricular point pressing can relieve xerostomia and insomnia in dialysis patients and improve their quality of life [199, 200].

**Fig. 8:**
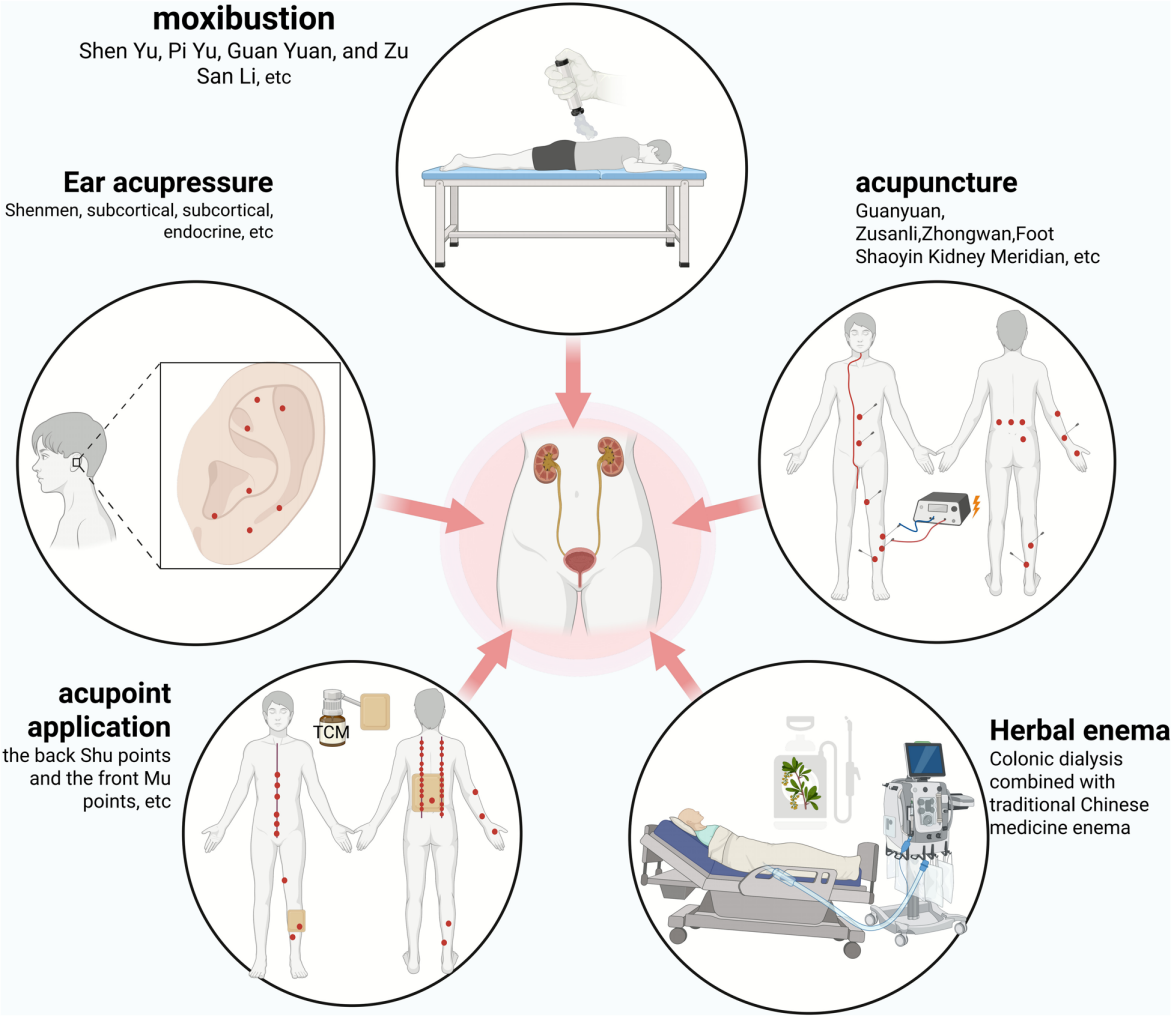
External therapies involving traditional Chinese medicine. External therapies of traditional Chinese medicine, such as acupoint application, auricular point therapy, acupuncture, moxibustion, and Chinese herbal enema, affect the progression of renal function by regulating lipid metabolism. These external therapies play important roles in improving the clinical symptoms of patients with kidney diseases

Various external traditional Chinese medicines can exert renoprotective effects and improve the clinical symptoms of patients with kidney diseases. Some studies have shown that they are related to regulating blood lipids and improving fatty acid metabolism, but most of the current research is limited to clinical studies, and the specific mechanisms involved have not been fully clarified. Future research should further clarify the specific molecular mechanisms by which external therapies improve kidney diseases and apply multiomics technologies such as lipidomics combined with systems biology methods to identify biomarkers and construct an overall regulatory network. Research should also focus on standardized intervention protocols and explore the synergistic effects of external therapies with modern drugs to scientifically promote the in-depth development and application of traditional Chinese medicine external therapies in the prevention and treatment of kidney diseases (Fig. 8).

## New delivery system for Chinese herbal medicine

Traditional Chinese herbal medicines have the potential to combat RF through multiple components and multiple targets, but they have limitations such as low bioavailability, poor targeting ability, and toxicity and side effects. The kidney has unique physiological structures such as the selective permeability of the glomerular filtration barrier, dense interstitial fibrous networks, and abundant vascular innervation, which not only provide specific targets for drug targeted delivery but also impose stringent requirements on the particle size, surface modification and biocompatibility of delivery systems. New drug delivery systems for Chinese herbal medicines (such as nanoformulations, liposomes, and micelles) can precisely match the pathological structural characteristics of the kidney to achieve targeted delivery, effectively regulate key pathological processes such as fatty acid metabolism, and inhibit the progression of RF in multiple dimensions. For example, chitosan-modified taxifolin liposomes, via the specific binding between the cationic properties of chitosan and anionic sites on the surface of renal endothelial cells, not only enhance drug absorption efficiency and bioavailability but also accurately target renal injury sites, reduce total cholesterol and triglyceride levels in diabetic mice, alleviate damage to the glomerular filtration barrier caused by lipid metabolism disorders, and thereby protect renal function [201]. New drug delivery systems for Chinese herbal medicines can also exert synergistic effects via dual mechanisms to alleviate RF. For example, the combination of deoxycholic acid-chitosan coated liposomes (DCS-Lips) and in-situ colon gels (IGE) reduces RF through two complementary approaches: improving oral bioavailability and regulating intestinal flora via enema [202]. Another study showed that encapsulating chlorophyll (Eug) and luteolin (Lut) in methacrylated hyaluronic acid (HAMA) hydrogel microspheres via microfluidic technology enables precise drug delivery and sustained release, enhances efficacy and safety, and protects the kidneys from hyperuricemic-induced damage [203]. Exosomes can serve as carriers for targeted delivery of Chinese herbs, providing a two-way path of "precision delivery plus efficacy enhancement and toxicity reduction" for the modernization of traditional Chinese medicines. For example, traditional Chinese medicine nanospheres are prepared from the decoction of *Aster tataricus*, and plant-derived exosomes are simultaneously extracted. Combined with interstitial injection technology, targeted delivery of drugs to the pulmonary interstitium via carpal tunnel injection can improve fibrosis, but its application in RF remains to be further developed [204].

**Fig. 9:**
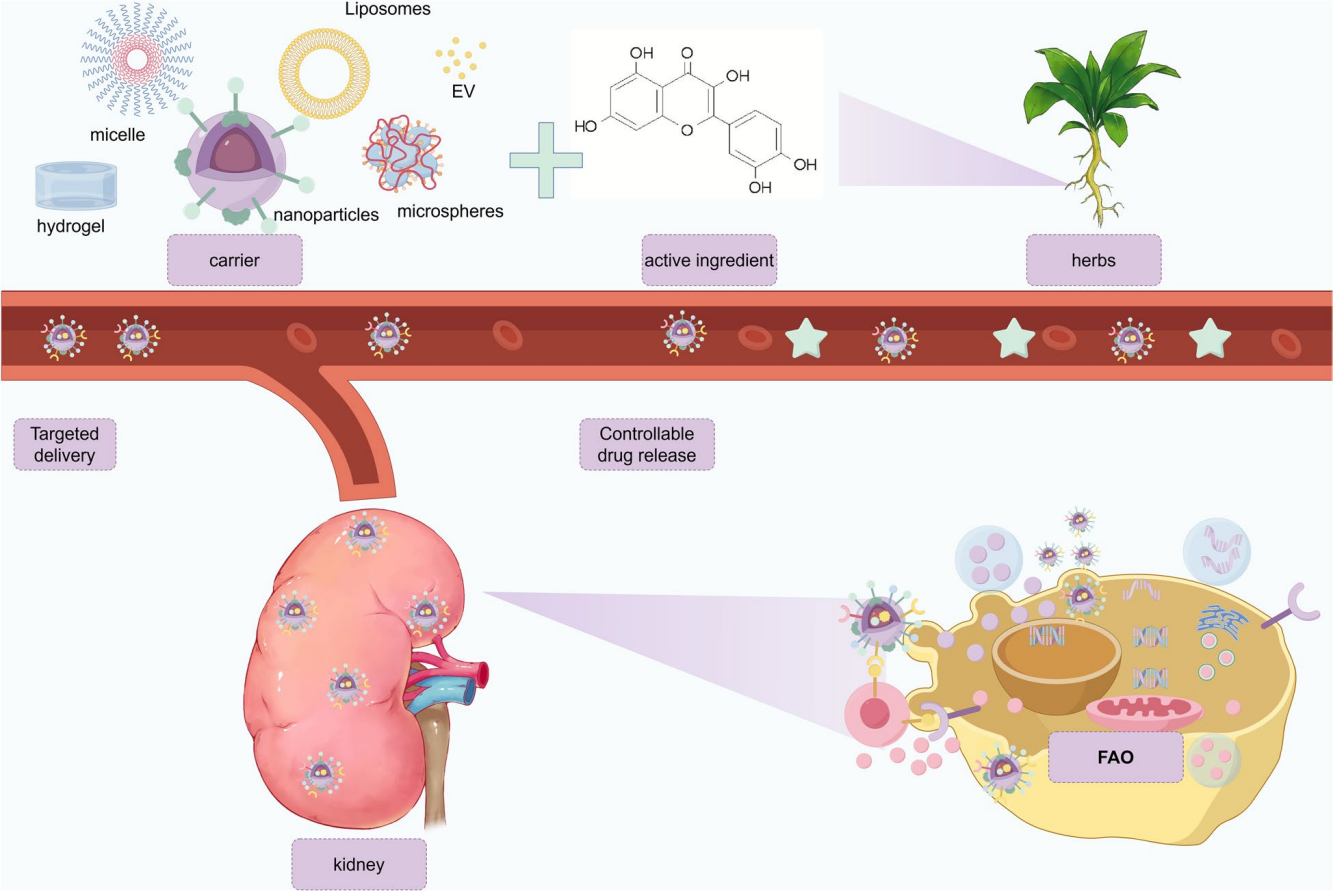
New delivery system for Chinese herbal medicine. The active ingredients of Chinese herbs can be delivered to the kidneys via nanoparticles, liposomes, micelles, hydrogels, nanospheres, and exosomes, among others, thereby affecting lipid metabolism and improving RF

The new drug delivery systems for Chinese herbal medicines not only retain the advantage of "holistic regulation" of Chinese herbal medicines but also integrate the precision of modern preparation technologies. They achieve precise intervention with low doses, avoid systemic side effects, and thereby provide a highly promising new strategy for the treatment of RF. However, challenges remain. The complexity of renal pathological structures increases the difficulty of carrier design; for instance, the size of the nano-core requires precise regulation—an excessively small core may be rapidly cleared, while an overly large one may fail to effectively penetrate renal tissues. Under fibrotic pathological conditions, reduced renal blood flow and vascular rarefaction further limit delivery efficiency. Moreover, renal cells exhibit heterogeneity, and current technical means cannot yet achieve precise and specific binding to RF-related cells, posing risks of increased adverse reactions or treatment failure [205]. The biocompatibility of some carrier materials needs to be improved; long-term administration may induce renal immune responses or exacerbate renal metabolic burden [206]. Furthermore, Chinese herbal medicines have complex components: the compatibility between multiple components and carriers, as well as synergistic release kinetics, are difficult to precisely regulate; in particular, some fat-soluble components may affect carrier stability, leading to premature drug leakage. Future efforts should focus on mechanism elucidation and carrier innovation. By combining the pathological characteristics of RF (e.g., changes in ECM composition and receptor expression), we should utilize intelligent nano-systems, integrate microenvironment-responsive drug release and multi-target intervention, enhance carrier biocompatibility and targeting precision, avoid potential renal injury, and accelerate their clinical translation (Fig. 9).

## Conclusion and outlook

The main pathological feature of RF is the excessive or disordered deposition of collagen-rich ECM, which leads to progressive damage to renal structure and function. Investigating the pathogenesis of RF and preventing disease progression are highly clinically important. Abnormal fatty acid metabolism plays an important role in the pathogenesis of RF. By summarizing articles related to fatty acid metabolism and RF, we conclude that the dysregulation of the PPARα/PGC-1α core axis initiated by miRNAs such as miR-21, miR-150, miR-9-5p, and miR-495, along with the drive of CPT, a key enzyme in fatty acid oxidation, mediates lipotoxic cascades. Additionally, the remodeling of the intrarenal immune-inflammatory microenvironment by gut microbiota-derived metabolites collectively promotes renal tubular cell dysfunction, impaired glomerular filtration function, and activation of myofibroblasts, thereby participating in the process of RF.

At present, clinical intervention for RF faces challenges. Therapeutic drugs still focus mainly on controlling the primary disease and using renin‒angiotensin system inhibitors, which have limited effects on reversing fibrosis. Intervention strategies targeting fatty acid metabolism (such as PPARα agonists and CD36 inhibitors) are still in the experimental stage and face challenges, including the complexity of target pathways, off-target toxicity of drugs, and individual differences in responses. Moreover, a single target faces difficulties in blocking the fibrosis network.

TCM holds distinct advantages in regulating fatty acid metabolism to ameliorate RF. Active ingredients of Chinese herbal medicine precisely target fatty acid metabolic pathways to exert anti-inflammatory, antioxidant and anti-apoptotic effects, restore mitochondrial function, and thereby alleviate RF. Chinese herbal compounds achieve multi-target synergistic regulation via compatibility combinations, compensating for the limitation that Western medicines targeting single molecules cannot address the complex fibrotic network. In addition, TCM external therapies and novel drug delivery systems integrated with modern technologies have further expanded the therapeutic dimensions for RF, laying a solid foundation for improving drug bioavailability and clinical efficacy. Under the guidance of the fundamental TCM pathogenesis of RF—"deficiency in origin and excess in superficiality"—and core therapeutic principles including "replenishing qi, activating blood circulation, and clearing turbid toxin", we summarize the commonly used TCM interventions for RF. Centering on three dimensions (Chinese medicinal active ingredients, Chinese herbal compounds and TCM external therapies), we integrate TCM pathogenesis with Western pathological mechanisms to elaborate the scientific connotation and material basis of TCM in treating RF. Studies have demonstrated that the TCM pathogenesis of "deficiency in origin and excess in superficiality" in RF is highly consistent with the Western pathological process mediated by abnormal fatty acid metabolism. The core TCM therapeutic principles of replenishing qi, activating blood circulation, and clearing turbid toxin can correspond to specific pathological targets, achieving a synergistic effect of treating both the root cause and superficial symptoms. Qi-replenishing Chinese medicines target the root cause of spleen-kidney qi deficiency by restoring the activity of the PPARα/PGC-1α pathway, improving mitochondrial function, promoting renal fatty acid oxidation, and inhibiting abnormal lipid uptake mediated by CD36. Blood-activating Chinese medicines address the superficial symptom of blood stasis obstructing collaterals through modulating pathways including TLR4/NF-κB, alleviating intrarenal inflammation induced by lipid accumulation, inhibiting collagen deposition, and unifying the pathological interventions of activating blood circulation to unblock collaterals with anti-inflammatory and anti-fibrotic effects. Heat-clearing and turbidity-purging Chinese medicines directly target the superficial symptom of damp-heat toxin pathogen via activating the AMPK/PPARα/PGC-1α axis, upregulating key fatty acid oxidation enzymes such as CPT1A, downregulating lipogenic factors including ACC and FAS, correcting lipid metabolism disorders, blocking the TGF-β1-Smad2/3 fibrotic pathway, and inhibiting lipotoxicity-mediated EMT.

Chinese herbal compounds target the intricate intertwined pathogenesis of deficiency in origin and excess in superficiality to achieve multi-pathway synergistic regulation of fatty acid metabolism and fibrotic networks. Moreover, TCM can rely on the holistic view of the gut-kidney axis to regulate intestinal flora for increasing short-chain fatty acids, indirectly modulating key regulators of renal lipid metabolism. This not only conforms to the TCM cognition that "the spleen is the acquired foundation of life and the spleen, intestine and kidney are interconnected", but also compensates for the limitation of single-target Western medicines, realizing systematic regulation of complex pathogenesis and pathological networks. TCM external therapies are based on the theory of correlation between meridians, collaterals and zang-fu organs, achieving synergistic intervention on pathogenesis and pathology via non-oral approaches, and realizing the organic integration of regulating zang-fu functions through meridians and maintaining metabolic homeostasis.

However, the current research has certain limitations. For example, Chinese herbal medicine (especially compound prescriptions) contain numerous chemical components, making it difficult to study their specific targets, action pathways, and mutual synergistic/antagonistic effects on fatty acid metabolism. Moreover, the processes of absorption, distribution, metabolism, and excretion of Chinese medicine components in the body are complex, and the effective active substances among their prototype components or metabolites, as well as their concentrations, are often unclear. The correlative research between TCM pathogenesis and Western pathological mechanisms remains superficial. Although it is currently established that the TCM pathogenesis of "deficiency in origin and excess in superficiality" in RF is closely associated with abnormal fatty acid metabolism, studies on the specific correlations between individual syndromes (e.g., spleen-kidney deficiency, damp-heat stasis-turbidity) and key fatty acid metabolism indicators (e.g., PPARα, CPT1A, lipotoxic products) are insufficient. A precise correlation system between syndromes and metabolic biomarkers has not yet been established. Challenges also exist between research models and clinical translation. The proportion of each medicinal herb in compound prescriptions and their interactions are difficult to precisely quantify and control, which increases the difficulty of standardization and mechanistic research. For future research, we need to further elucidate the mechanisms of Chinese herbal monomers and compounds, clarify the metabolic profiles of active components and the network targets through which multi-components synergistically regulate fatty acid metabolism, and establish a standardization system for compound prescriptions. We should also construct precise correlations between TCM syndromes of RF and fatty acid metabolic biomarkers to achieve the organic integration of treatment based on syndrome differentiation and targeted intervention. Additionally, it is necessary to strengthen high-quality clinical research, promote standardization and modernization, explore the precise treatment model integrating disease and syndrome differentiation, and, through in-depth interdisciplinary integration, innovate new drug research, promote the modernization of traditional Chinese medicines, and accelerate clinical translation to provide new progress and breakthroughs for the treatment of RF.

## Data Availability

No datasets were generated or analysed during the current study.

## Abbreviations

ACC: Acetyl-CoA carboxylase
CD36: Cluster of differentiation 36
CKD: Chronic kidney disease
CoA: Acyl coenzyme A
CPT1A: Carnitine palmitoyltransferase 1α
DN: Diabetic nephropathy
ECM: Extracellular matrix
EMT: Epithelial‒mesenchymal transition
ESRD: End-stage renal disease
ESRRA: Estrogen-related receptor alpha
FAO: Fatty acid oxidation
FAS: Fatty acid synthase
MF: Myofibroblasts
PGC-1α: Peroxisome proliferator-activated receptor γ coactivator 1α
PPARα: Peroxisome proliferator-activated receptor α
RF: Renal fibrosis
SCFAs: Short-chain fatty acids
SIRT1: Sirtuin 1
STAT6: Signal transducer and activator of transcription 6
TCM: Traditional Chinese medicine
TWIST1: Twist-related protein 1
